# Does regular engagement with arts and creative activities improve adolescent mental health and wellbeing? A systematic review and assessment of causality

**DOI:** 10.1016/j.ssmph.2025.101845

**Published:** 2025-07-22

**Authors:** Sam Hugh-Jones, Stephanie Ray, Anna Wilding, Matt Sutton, Neil Humphrey, Luke Munford

**Affiliations:** a Health Organisation, Policy and Economics, University of Manchester, Manchester, UK; b Manchester Institute of Education, University of Manchester, UK

**Keywords:** Adolescent mental health, Arts and cultural engagement, Arts and creative activities, Causal systematic review, Bradford hill

## Abstract

**Background:**

There is a growing body of evidence on the relationship between arts and creative activities and adolescent mental health and wellbeing. However, most research has focused on short, sharp creative arts interventions, and not on regular, day-to-day engagement with arts and creative activities. It is unclear the extent to which this complex relationship can be considered causal. This systematic review aimed to summarise the quantitative evidence on engagement with arts and creative activities and assess whether it supports causal conclusions.

**Methods:**

We systematically searched 10 databases. We included any quantitative studies (cross-sectional and longitudinal observational studies, natural and quasi-experimental designs, and controlled trials) assessing the link between regular engagement (multiple instances over a period of more than one week) with arts and creative activities, and adolescent (10-19y) mental health and wellbeing. We included studies published in English from 2014 to 2024. Methodological quality was assessed using JBI critical appraisal tools. Support for causal conclusions was assessed using the Bradford Hill Viewpoints.

**Results:**

Of 7769 records screened, 28 were selected for inclusion. Most studies suggested a positive association between arts engagement and adolescent mental health and wellbeing. We found some support for all Bradford Hill viewpoints, but less support for experimental evidence and a dose-response relationship.

**Discussion:**

This review provides moderate support for a causal relationship between arts engagement and adolescent mental health and wellbeing. More evidence from randomised experiments or generated by applying causal inference methods to observational data is needed to better account for selection, confounding, and reverse-causality.

## Highlights

• Regular arts engagement is linked to better adolescent mental health.
• We systematically reviewed causal evidence for this.
• The evidence base provides some support for causal conclusions.
• Evidence is particularly strong for music and internalising symptoms.
• More experimental/quasi-experimental research is needed.

## 1 Introduction

Adolescent mental health and wellbeing (MHWB) is in decline globally (Blanchflower et al., 2024), which is increasingly being recognised as a ‘crisis’ (Gunnell et al., 2018). There is evidence that engaging with arts and creative activities may be beneficial for adolescent mental health (Fancourt et al., 2023), including in low-income communities around the world (Bux & van Schalkwyk, 2024; Golden et al., 2024). However, public funding and provision of arts in many European countries, as well as the USA and UK, has declined in recent years (Compendium of Cultural Policies and Trends in Europe, 2019; Stubbs et al., 2022; Easton & di Novo, 2023; International Council of International Committee of Museums, 2025), especially in schools (Evennett, 2021). Mental health concerns are often overlooked in low- and middle-income countries (Erskine et al., 2017), and they may be particularly well-placed to benefit from leveraging existing culturally-relevant practices (Frasco et al., 2025; Golden et al., 2024).

The literature base examining the relationship between arts engagement and adolescent mental health and wellbeing continues to grow. Recent reports have summarised a portion of the existing evidence (Bone & Fancourt, 2022; Fancourt et al., 2023). Other recent reviews focused specifically on arts-based mental health interventions (Williams et al., 2023), including in low-income communities globally (Bux & van Schalkwyk, 2024). However, to the best of our knowledge, the most recent review article that included regular arts engagement was conducted in 2017 (a rapid review by Zarobe & Bungay, 2017), and the last systematic review in 2008 (Daykin et al., 2008). Furthermore, these past reviews often conflated regular arts engagement, interventions, and art therapy. Cursory searches indicated that a large amount of new research has been published since Bone and Fancourt (2022) and Fancourt et al.’s (2023) overviews, let alone since previous systematic reviews. Therefore, a systematic review of this evidence base is warranted to provide a stronger understanding of the causal relationship between engaging with arts and creative activities and mental health, to inform policy and commissioning decisions around arts for young people. Particularly given notable declines in public funding for young people's arts, it is also important and policy-relevant to be able to evaluate the extent to which the evidence base can support causal conclusions, and where it may be lacking.

### 1.1 Adolescent mental health and wellbeing (MHWB)

There are many conceptualisations of ‘mental health’ and ‘wellbeing’. The ‘two continua’ model (Westerhof & Keyes, 2010) brings together the definitions of ‘good mental health’ as an absence of mental health conditions (such as anxiety and depression), and of ‘wellbeing’, which encompasses ‘emotional wellbeing’ (life satisfaction and happiness), ‘psychological wellbeing’ (self-realisation and positive functioning), and ‘social wellbeing’ (positive feeling of social value). In young people's words, wellbeing is about “feeling good, feeling that their life is going well, and feeling able to get on with their daily lives” (Anna Freud, 2016, p. 6).

It is increasingly understood and accepted that MHWB, particularly in adolescents and young people, has declined in recent years, and this worsening of young people's mental health can be seen around the world, including the UK, Spain, Italy, Sweden, France, the Netherlands, Germany, and New Zealand (Blanchflower et al., 2024). Estimates suggest that 50 % of these problems have an onset prior to age 14 (Kessler et al., 2005).

This is important to policymakers and practitioners alike, not just because of the direct effects on young people, but also due to the increasing costs of treatment, and pressure on already struggling youth mental health services (Hall, 2023), as well as negative effects on the wider economy (Knapp et al., 2016). It is increasingly understood that prevention and early intervention to protect and promote adolescent MHWB is key to reducing costs, and safeguarding future adult outcomes for young people (Goodman et al., 2015).

### 1.2 Arts and creative activities and adolescent mental health

In recent years, there has been significant interest in ‘creative health’; the role that arts and creative activities (such as visual and performing arts, crafts, film, literature, and many more) can play in promoting health and wellbeing (Creative Health Review, 2023). The 2023 Creative Health review from the UK highlighted the robust evidence for the effectiveness of creative health approaches for health and wellbeing, in conjunction with medical treatment, but also in their own right. Whilst ‘creative health’ encompasses many ways of connecting arts, creativity, and health, one particular strand involves exploring the health and wellbeing benefits of engaging with arts and cultural activities regularly, as part of day-to-day life. It has been suggested that arts-based strategies may be able to support youth mental health globally, due to the ability to leverage existing, culturally-relevant practices (Golden et al., 2024). In particular, there is a growing body of research on the relationship between engagement with arts and creative activities and MHWB, including in adolescents. This engagement may be as part of the school curriculum, in-school extra-curricular activities, or in their free time. In addition, engagement may be ‘active’ or ‘receptive’. ‘Active’ engagement generally involves making, creating, or partaking in an artistic activity (e.g. drawing, painting, writing, performing), whereas ‘receptive’ engagement generally involves interacting with art that someone else has created (e.g. viewing, listening to, discussing, critiquing) (Davies et al., 2012).

Bone and Fancourt (2022) and Fancourt et al. (2023) have summarised a large portion of the evidence base on arts engagement and adolescent MHWB, but not using systematic methods. Their findings suggest that for young people, arts engagement (both active and receptive) was associated with increased self-confidence, greater development and appreciation of self-identity, and increased self-esteem. Bux and van Schalkwyk (2024) reviewed arts-based programs (although not regular engagement) in low-income communities, finding positive impacts on self-acceptance, autonomy, mastery, and positive relationships. Williams et al. (2023) conducted a systematic review of participatory-arts-based programs for promoting mental health and wellbeing in young people across various settings around the world. Their findings suggested that these programmes were linked with improved wellbeing and quality of life, and increased self-esteem. Williams et al. (2023) also explored the mechanisms by which participatory arts-based programmes may affect youth MHWB. They identified themes such as creating a protected space away from everyday life, fostering connection and team spirit with other young people, and creating a more positive self-narrative through expression and exploration of emotions, as well as different perspectives and identities. Fancourt et al. (2023) also identified a number of mechanisms, including improved self-esteem, self-control, and emotional regulation.

These existing reviews and reports noted a number of limitations to the current evidence base. In particular, Bone and Fancourt (2022) highlighted that much of the existing literature focuses on “specific arts-based interventions”, and that a greater research focus is needed on the effects of “ubiquitous engagement in the arts as part of daily life” (p.27). They also noted a lack of methods that can key issues in this relationship; it is likely affected by bi-directionality (i.e. MHWB and arts engagement likely both influence each other), confounding on other key characteristics (e.g. socioeconomic status has been shown to affect both arts engagement and MHWB; Mak & Fancourt, 2021; Mac-Ginty et al., 2024), and selection. Without methods that can address these, the extent to which the relationship between engagement with arts/creative activities and adolescent MHWB can be considered causal is unclear.

### 1.3 Causality and the Bradford-Hill viewpoints

Establishing causality is a key factor to ensure that research is policy-relevant (Kim, 2019). It may be of particular importance in relation to arts and mental health, as the relationship is complex and likely bi-directional, as well as being confounded by a wide range of individual and social factors (Wang et al., 2020).

In 1965, Austin Bradford Hill set out nine ‘viewpoints’; characteristics that we may want an observed association to have in order for it to have some level of ‘causal’ interpretation (we shall henceforth refer to these as ‘BH viewpoints’). These were very influential and are still widely used (Stewart, 2020). Despite originating in epidemiology, they have now been applied to questions in psychiatry (Moore et al., 2017), behavioural science (Norman et al., 2016), and health economics (Pickett & Wilkinson, 2015; Boniface et al., 2017). Table 1 summarises the BH viewpoints (Hill, 1965).

**Table 1:** Bradford Hill viewpoint definitions (Hill, 1965).

BH Viewpoint	Definition
Strength	The observed association should be large in magnitude
Consistency	The association should be repeatedly observed across places, settings, and times
Specificity	The outcome should be associated with only the specific exposure
Temporality	The exposure should be observed at an earlier point in time than the outcome
Biological gradient	There should be a clear dose-response relationship between exposure and outcome (i.e. greater exposure is related to greater outcome)
Plausibility	The association should be plausible given existing knowledge of mechanisms
Coherence	The association should not seriously conflict with existing evidence
Experiment	Evidence should come from experimental designs and settings
Analogy	There should be evidence linking similar exposures and similar outcomes

These viewpoints can be easily adapted into criteria for use in evaluating the causal support for hypotheses in a body of evidence (Khan et al., 2012; Stewart, 2020). This framework has increasingly emerged as a technique for establishing causality in an evidence base (Boniface et al., 2017), and many systematic reviews have now used these viewpoints to assess the extent to which a body of evidence can support causal interpretations (Shimonovich et al., 2022). Use of the BH viewpoints is recommended by the Joanna Briggs Institute (JBI) when considering the assessment of causality in systematic reviews of etiological (association) studies (Moola et al., 2015).

The viewpoints are not necessarily equal in their contributions to conclusions of a causal relationship. Temporality is often considered to be the most important, along with experimental evidence, given that they rely less on the subjective judgement of a researcher or reviewer (Stewart, 2020). The other viewpoints also assist in drawing causal conclusions, but may not be necessary. In particular, a weak or non-specific association may still be considered causal (Hill, 1965). Many causal reviews assess only a number of viewpoints, combining or dropping viewpoints that they deem unnecessary (Shimonovich et al., 2022). There are a wide variety of ways in which these viewpoints have been operationalised and applied, especially in an era with more data and advanced statistical methods (Fedak et al., 2015). The specific definitions of the viewpoints and applications in this review are outlined in Section 2.5. and Appendix 2.

### 1.4 The present study

Given the large and growing evidence base on arts engagement and adolescent mental health, and the policy relevance of these findings, this study sought to systematically review the body of evidence and assess the causality of findings.

We investigated ‘regular’, everyday arts engagement in particular, as opposed to specific and time-limited targeted interventions for those at risk of poor mental health, as this has been highlighted as an area that may require more research (Bone & Fancourt, 2022), and the latter already has an established body of evidence (O'Donnell et al., 2022). We note that the literature also uses the terms ‘ubiquitous’, ‘day-to-day’, and ‘everyday’ to address this concept, with the common theme of engaging often and over an extended period of time, in contrast with short, intensive interventions or programs. This review uses the term ‘regular’ to encompass this idea, although we acknowledge that none of these terms perfectly individually capture this type of engagement. We also consider universal provision (e.g. in schools) to meet the criteria for ‘regular’ engagement, and programs that would encourage young people to engage a number of times over, for example, a number of weeks.

#### 1.4.1 Research questions

The primary aim of this review was to collate and synthesise all the existing evidence that links regular engagement with arts and creative activities and adolescent mental health and wellbeing. We aimed to answer the following primary research question.

1. What is the evidence on the associations between regular engagement with arts and creative activities and adolescent mental health and wellbeing?

Our secondary aim was to investigate to what extent this evidence represents a causal effect, as opposed to an association.

2. To what extent does the accumulated evidence base (and underpinning theory) support the fact that the relationship between regular arts engagement and creative activities and adolescent mental health is causal?

Table 2 summarises RQ1 in the PEO (Population, Exposure, Outcome) framework, as this is more appropriate to reviews of association than the traditional PICO (Population, Intervention, Comparator, Outcome) framework (Moola et al., 2015).

**Table 2:** PEO framework for RQ1.

	Description
Population	Adolescents (Age 10–19, based on the WHO definition of adolescence: World Health Organisation, n.d.)
Exposure	Some level of ‘regular’ engagement with arts and creative activities (compared to less or none)
Outcome	Mental health/wellbeing

## 2 Methods

This review was performed according to Preferred Reporting Items for Systematic Reviews and Meta-Analyses (PRISMA) guidelines (Page et al., 2021), the checklist for which can be found in Appendix 1. The review protocol was registered on PROSPERO (CRD42024610518). Minor deviations from the original protocol are outlined in Appendix 3.

### 2.1 Search strategy

We searched the following databases to identify all literature relevant to our research question.

• Applied Social Sciences Index and Abstracts (ASSIA)
• British Education Index (BEI)
• Child Development and Adolescent Studies (CDAS)
• CINAHL
• EconLit
• Education Resources Information Centre (ERIC)
• PsycINFO
• PubMed Central
• RILM Abstracts of Music Literature
• Web of Science

Search terms were informed by previous similar systematic reviews (Daykin et al., 2008; Viola et al., 2024; Williams et al., 2023; Zarobe & Bungay, 2017), and input from an information specialist. An example search strategy can be found in Appendix 4.

### 2.2 Study selection

Identified records were assessed according to the inclusion and exclusion criteria in Table 3.

**Table 3:** Study inclusion and exclusion criteria.

Inclusion Criteria	Exclusion Criteria
Quantitative empirical studies, including journal articles, pre-prints, working papers, and Doctoral dissertations	Qualitative studies, reviews, research protocols
Studies in English	Studies not in English
Published 2014–2024	Published before 2014 or after 2024
Studies with a mean age of subjects between 10 and 19 (both	Mean age of subjects not within ages 10–19 (either at exposure or outcome)
exposure and outcome)	
Include some measure of engagement/availability of arts and creative activities OR assesses a	Targeted programs e.g. those aimed at specific groups
universal program	
that may increase arts engagement	
for whole study population (e.g.	
whole school)	
Arts engagement must be ‘regular’ (on more than one distinct occasion over the course of more than one week)	Studies where arts engagement is one-off (e.g. a one-day event)
Comparator is less or no engagement	Studies where arts engagement is not distinct from other activities (e.g. total extra-curricular engagement)
Include some measure of mental health/wellbeing (any measure	
with some level of validity/reliability information, or objective measures e.g. substance	
abuse)	
Reports some measure of association between arts	
engagement and mental health/wellbeing	

Adolescence was defined according to the WHO's definition (ages 10–19; World Health Organisation, ND). For the purpose of this review, ‘regular’ arts engagement was defined as any engagement with an arts or creative activity on more than one distinct occasion, over an extended (greater than one week), regular (weekly, monthly, etc.) or indeterminate^1^ (not in the case of programs) time period, including programs that provide universal access to arts activities for the population of interest (e.g. a whole school). Operationally, this meant excluding studies that investigated arts and creative programs or interventions that are one-off (e.g. a one-week intensive program or camp). We also excluded programs targeted at a specific group (e.g. demographic, at-risk, diagnosed with a mental illness), as these would likely have different effects than on our population of interest (all adolescents). Keywords were chosen to reflect the breadth of potential arts and creative activities, but all activities that could reasonably be considered to fit this definition were eligible. These were based on past reviews, as well as classifications outlined by Davies et al. (2012), and include both active engagement (creating/making/participating) and passive engagement (watching/listening/consuming). Studies were included if the comparator was less or no arts engagement, even if multiple forms of arts were investigated. Where studies only compared one activity with another (e.g. another arts activity or sports), they were excluded, unless they also compared these activities with no engagement. The timeframe (2014–2024) was chosen as we wanted to review contemporary evidence in this field. 2014 represents 10 years prior to the point at which this search was undertaken. Furthermore, all of the work on young people cited by Fancourt et al. (2023), and the vast majority by Bone and Fancourt (2022), as well as their novel analyses, fell within this timescale, indicating that restricting the search to this period would include all of this recent, relevant literature. We also note that this period has considerable crossover with the last published review by Zarobe and Bungay (2017), which itself was based on a 2008 review by Daykin et al.

Eligible outcomes included any measures of constructs or domains associated with mental health or wellbeing, including (but not limited to) stress, self-harm, self-esteem, resilience, confidence, and quality of life. Manifestations of externalising behaviour, such as antisocial behaviour and substance abuse, were also included. These were chosen to reflect the ‘two continua’ model of mental health (Westerhof & Keyes, 2010), and the breadth and concepts encompassed in ‘mental health’ and ‘wellbeing’ (Black et al., 2023; Khanna et al., 2024).

### 2.3 Screening procedure

Rayyan software (Ouzzani et al., 2016) was used for the title/abstract and full-text screening processes. A screening tool was developed and pilot tested by four authors on a small sample of records to refine inclusion criteria and ensure clarity. At both stages, a portion of records were independently screened by two reviewers (1 % of records at abstract and 10 % at full text stages). Discrepancies were resolved by discussion and one reviewer screened the remaining records.

At the title/abstract screening stage, there was 100 % agreement (Cohen's k = 1) for the sub-sample of dual-screened records. At full-text screening, there was ‘substantial agreement’ (Cohen's k = 0.64; McHugh, 2012).^2^ At this stage, disagreements were discussed, resolved, and re-screened, and screening was continued by one reviewer.

### 2.4 Critical appraisal

Methodological quality was assessed using the revised JBI critical appraisal tools relevant to each study design: analytical cross-sectional, cohort/longitudinal, quasi-experimental, and RCT (JBI, 2020a; JBI, 2020b; Barker et al., 2023; Barker et al., 2024). Two reviewers appraised 10 % of included studies, and following discussion of results, one reviewer appraised the remaining studies. Studies were assessed on each checklist item as ‘yes’, ‘no’, or ‘somewhat’ (/’unsure’), and were awarded 1, 0.5, or 0 points respectively. Studies were then rated as ‘high’ (>75 %), ‘moderate’ (50–75 %), or ‘low’ (<50 %) quality, based on the percentage of applicable available checklist items.

Certainty of evidence was assessed in line with guidelines of how to apply GRADE criteria in the absence of meta-analysis (Murad et al., 2017).

### 2.5 Causal evaluation

The Bradford Hill (BH) viewpoints (Hill, 1965) were applied to assess the extent to which causal conclusions could be drawn from each included study, and from the body of evidence as a whole. Where studies assessed the relationship between arts engagement and multiple outcomes, criteria were applied to each result (exposure-outcome pair). These criteria have been applied in different ways across many different systematic reviews (Shimonovich et al., 2022). Table 4 presents the six BH viewpoints applied in this review, and the way in which they are applied. As with a number of past causal reviews (Biddle et al., 2019; Norman et al., 2016; Stahl et al., 2013) we combined the plausibility and coherence viewpoints as they largely both consider very similar concepts. We re-characterised biological gradient as dose-response, as it is more applicable to our setting (Fedak et al., 2015; Norman et al., 2016). We expanded experimental evidence to include natural and quasi-experimental evidence (Craig et al., 2025), as we expected to find little in the way of true experimental evidence, given the difficulty in randomising arts engagement, and ubiquity of observational data. We dropped the viewpoint of specificity, as it seems unlikely that a specific relationship would be observed in the case of our question; there are many factors that can affect MHWB (Sawyer & Patton, 2018), and arts engagement may impact many outcomes other than just MHWB (Karkou et al., 2022). This has often been done in past causal reviews (Fedak et al., 2015; Mente et al., 2009; Norman et al., 2016), especially where mental health is an outcome (Moore et al., 2017). Analogy is one of the least-used of the BH viewpoints (Shimonovich et al., 2022), in part due to its nature as a highly subjective judgement (Stewart, 2020), which is “largely driven by the creativity of the investigators” (Mente et al., 2009, p. 661). Indeed, Bradford Hill himself describes it as the weakest form of evidence. Therefore, we also chose not to evaluate this viewpoint.

**Table 4:** Application of BH criteria and level of application.

Viewpoint	Definition in this review	Level applied at (Result or body of evidence (BoE)
Strength	Statistically significant association, followed by effect size	Result
Temporality	Is temporal order accounted for? I.e. are measures of arts engagement taken from before outcome measures?	Result
Dose-response (biological gradient)	Is there evidence that more/less exposure is associated with better/worse mental health?	Result
Experimental/Quasi-Experimental Evidence	Do findings come from experimental or quasi-experimental evidence? If not, do studies use statistical methods to account for confounding?	Result
Plausibility/Coherence	Are there credible mechanisms for effect? Is there any evidence for these mechanisms?	BoE
Consistency	Significant associations with consistent directions found across settings, populations, methodologies	BoE

Where viewpoints were applied at result level, support for each viewpoint was assessed as ‘strong’, ‘moderate’, or ‘weak’.^3^ Technical explanations of the application of these viewpoints are presented in Appendix 2.

We note here that although we assess methodological quality and causal support separately, they are not entirely discrete constructs. Aspects such as temporality are common to both. Therefore, we anticipated a likely correlation between methodological quality and causal support (although perhaps less so for cross-sectional designs, as it is entirely possible to have a high-quality cross-sectional study that provides little support for causal conclusions).

### 2.6 Analytic strategy

Given the heterogeneity of the included studies with regards to exposures, outcomes, and methodologies, data were analysed through narrative and visual synthesis. In particular, graphical synthesis (such as adapted harvest plots; Ogilvie et al., 2008) allowed for effective summary of the multiple aspects of evaluation (methodological quality and causal evaluation) used in this study. Meta analysis was considered, but due to heterogeneity in methods, exposures and outcomes, was deemed not possible, as is often the case in systematic reviews of associations (Moola et al., 2015).

## 3 Results

### 3.1 Search results

**Fig. 1:**
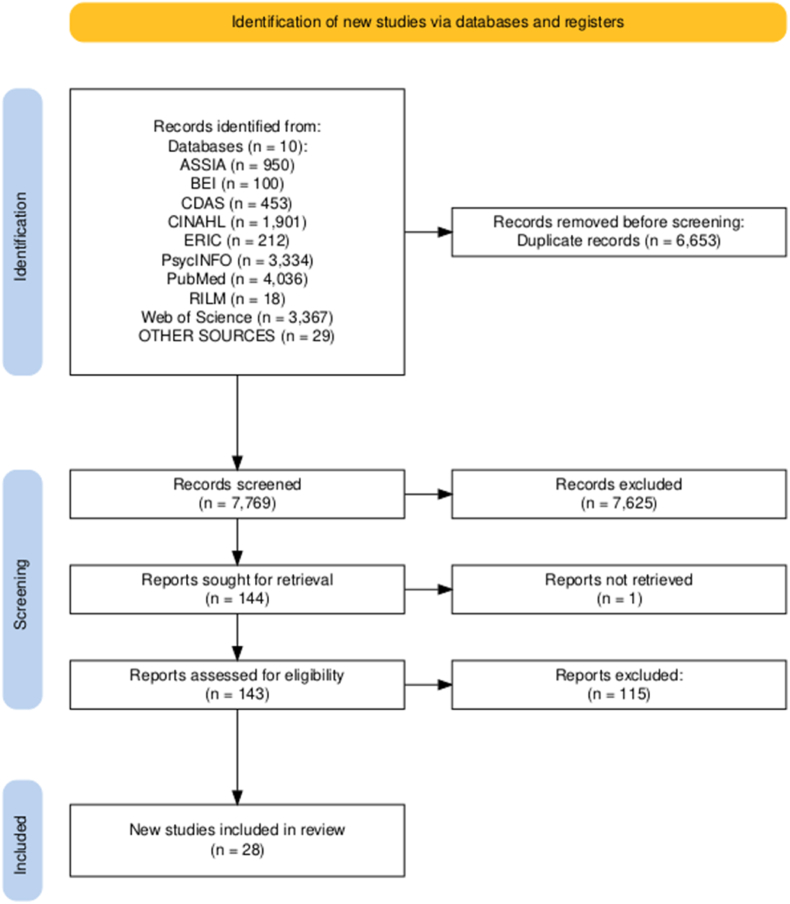
PRISMA screening and study selection flowchart ***Note:***Fig. 1 was generated using the tool created by Haddaway et al. (2022). EconLit was also searched but our search strategy identified no studies.

Searches identified a total of 14,400 articles. After de-duplication, we screened 7769 titles and abstracts against inclusion criteria. A total of 144 records were then retrieved for full-text review. Of these, 28 met the inclusion criteria and were selected for inclusion in this review. The most frequent reasons for exclusion at this point were wrong age (n = 19), arts/creative engagement not distinct from other activities (n = 17), and targeted interventions (n = 17). 25/28 included studies were published academic journal articles, and the remaining three were doctoral dissertation chapters. Analysis was undertaken at the individual result level, which constituted 56 exposure-outcome pairs across the 28 studies (as many studies investigated multiple MHWB outcomes or arts activities). Fig. 1 presents the study selection process.

### 3.2 Characteristics of included studies

Table 5 presents the characteristics of the 28 included studies.

**Table 5:** Characteristics of included studies.

Authors and Year	Country	Setting	Specific population of Interest	Sample Size {at time points} (%female)	Age range {at time points}	Study design	Description of arts engagement	Arts category	MHWB outcomes	Outcome measures	Key findings
CROSS-SECTIONAL STUDIES											
Badura et al. (2015)	Czech Republic	Nationally representative cohort		10,503 (50.8 % F) 3332 {age 11} 3541 {age 13} 3630 {age 15}	11, 13, 15 (mean 13.1y)	Cross-sectional	Free-time organised activity participation. Responses clustered to: 'All rounders', 'artists', 'individual sports', 'team sports', 'inactive'	Multiple/not separated	Life satisfaction Feeling low Irritable/Bad tempered Nervous Difficulty sleeping	LS: ‘Here is a picture of a ladder. The top of the ladder ‘10’ is the best possible life for you and the bottom ‘0’ is the worst possible life for you. In general, where on the ladder do you feel you stand at the moment?’ Others: ‘In the last 6 months: how often have you had the following … ?’ with 5 response categories ranging from about every day to rarely or never. Responses dichotomized using a “generally accepted cut-off set to at least once a week"	Being an 'artist' associated with higher LS than 'inactive', but not other outcomes. Effect of being an 'artist' on other outcomes differs by age and gender, generally worse for males and 15y vs 11y
Clarke and Basilio (2018)	UK	KS4 and KS5 in one school in NW England		275 201 {KS3} 74 {KS4}	11-12 {KS3} 15-16 {KS4}	Cross-sectional	Creative engagement in performing arts' scale, created from other scales (enjoyment and harmonious passion, intention to take any performing arts GCSE) - KS3 Currently studies any performing arts GCSE - KS4	Other	Wellbeing	McLellan & Steward (2015) wellbeing instrument - subdivided into interpersonal, ife satisfaction, competence, negative emotion	Y7 'PA engaged' pupils had worse intepersonal and competence wellbeing Y10 'PA engaged' pupils had better life satisfaction wellbeing
da Silva et al. (2023)	Brazil	Online survey of legal guardians during COVID-19		517 (47 % F)	7-18 (mean 11.5, SD 3.16)	Cross-sectional	Time spent listening to music on weekdays and weekends	Music (listening)	Inattention/hyperactivity	Brazilian version of the Child Behavior Checklist for ages 6–18 (CBCL/6–18) [only attention and ADHD subscales used]	Listening to more music as a media behaviour associated with fewer attention problems and ADHD symptoms
Deer et al. (2023)	China	Secondary schools in Guizhou		2139 (55.3 % F)	13-17 (mean 14.3)	Cross-sectional	'Art participation' A few sample items are: “I will express the beauty artistically found in my life.”, “I often go to art exhibitions or community cultural activities.”, “I attend all kinds of courses related to art.”, “I like to photograph natural landscapes, buildings, and figures with aesthetic and artistic feeling." (Full description not given)	Multiple/not separated	Self-efficacy	Schwarzer & Jerusalem (1995) instrument	Art participation linked to increased self-efficacy, mediated by cognition and emotion
Dorris et al. (2022)	USA	Nationally representative cohort	7th graders	3675	7th graders (12–13)	Cross-sectional	Going to the symphony, playing a musical instrument over the summer, or participating in a music group in school.	Music(both)	Self-esteem	Sum score of six Likert-scale items, including prompts such as “I have a positive attitude toward myself.”	Positive but non-statistically significant association between music engagement and self-esteem
Gottfried (2021)	USA	Nationally representative cohort		2424 (45.8 % F)	12th Grade (17-18y)	Cross-sectional	Student involvement in: “school newspaper or yearbook” “music or other performing arts”.	Other (Yearbook) Music (playing)	Depression	Multiple 5-point likert statements mapped onto DSM-5 symptoms	Involvement in school newspaper or yearbook associated with lower depressive symptoms, but performing arts associated with higher symptoms. Associations found mostly in males.
Kennewell et al. (2022)	Australia	Sout Australia, school years 4–9	Low socio-economic status nackgrouns	61,759 (49.0 % F)	8-14y	Cross-sectional	After school activities: Music lessons or practice Arts and crafts	Music (playing) Arts and crafts	Wellbeing: Happiness Sadnesss (reversed) Worry (reversed) Emotional regulation Life satisfaction Engagement Optimism Perserverance	Happiness, engagement, and perseverance: three items each from the EPOCH Measure of Adolescent Well-Being. Sadness and optimism: three items each from the Middle Years Development Instrument. Emotion regulation: three items from the Emotion Regulation Questionnaire for Children and Adolescents. Life satisfaction: five items from the Satisfaction with Life Scale for Children. Worry: four items developed by the SA Dept Ed and the Telethon Kids Institute	Some associations between frequency of engagement with music or arts and crafts after school and higher wellbeing scores in some domains. Music engagement also associated with lower scores in other domains (happiness, emotional regulation, life satisfaction).
Mak and Fancourt (2019)	UK	Nationally representative cohort		6209 (50.1 % F)	11y	Cross-sectional	How often child: (1) listen to or play music, (2) draw, paint, or make things, and (3) read for enjoyment, not for school but as an extracurricular activity at home	Music(both) Arts and crafts Other	Self-esteem	Bespoke survey instruments that draw on Rosenberg's 10-item self-esteem scale	Participating in arts 'most days' associated with greater SWB compared to any other category, and even more so (effect doubled) when compared to rarely/never participating.
Miranda (2022)	Canada	University		647 (76.8 % F)	17-21y (mean 18.8, SD 0.93)	Cross-sectional	Being a 'musician' Playing an instrument Amount of time per week spent playing musical instrument Amount of years played musical instrument regularly Years of musical traininhg through private lessons Years of musical training at school Electronic music making	Music (playing)	Internalising symptoms	Kessler Psychological Distress scale (six-item, likert 1–5)	No difference in internalising symptoms by musicianship status. Small correlation between music playing and music education and increased internalising symptoms
Oosterhoff et al. (2017)	USA	Nationally representative cohort		10148 (51.1 % F)	13-18y (mean 15.2, SD 1.51)	Cross-sectional	Duration of involvement in: band/orchestra/chorus student newspaper/yearbook	Music (playing) Other (Yearbook)	Self-esteem Substance use	5 items adapted from Rosenberg self-esteem scale Frequency that they (1) had at least 1 drink in the past 12 months (2) and used marijuana/hash in the past 12 months on a 6-point scale from 1 (never) to 6 (nearly every day). Free-response report of the number of days that they smoked at least 1 cigarette, cigar, or pipe in the last 12 months	Greater duration of music involvement was indirectly associated with lower substance use and greater self-esteem, through greater teacher support
Pongutta and Vithayarungruangsri (2023)	Thailand	Public primary school grades 4-6		2277 (56.6 % F)	9-10y - 802 11y - 753 12y - 645 >12y - 77	Cross-sectional	Time spent doing: Art (drawing, painting, and sculpturing) Music (listening to music, singing, and playing musical instruments)	Arts and crafts Music (both)	Life satisfaction	(“not at all (0 point)” to “totally satisfied, (10 points)”) [From Children's World Project]	High hours of music engagement per day associated with worse life satisfaction than no music engagement
Ros-Morente et al. (2019)	Spain	Education centres which provide band/orchestra/choir, in Valencia and Catalonia	Musicians and non-musicians	1315 (62.2 % F)	Not stated, 'adolescent'	Cross-sectional	Membership of youth bands/choirs	Music (playing)	Emotional development (emotional awareness, emotion regulation, emotional autonomy, social competence and life competences) Satisfaction with life	Emotional Develpoment Questionnaire Satisfaction with Life Scale	Musicians displayed statistcially significantly higher emotional competencies than non-musicians (highest for emotional awareness) Also significantly higher life satisfaction
Yıldırım et al. (2024)	Turkey	Youth centre in a province		623 (34.7 % F)	11-12y 11 (47.5 %) 12 (52.5 %)	Cross-sectional	Participating in 'artistic activities' at youth centre (music, folk dances, painting and marbling courses) - twice a week, 2h per session	Multiple/not separated	Digital Addiction Psychological resilience Aggression (reactive, proactive, total)	Digital Addiction Scale CAPRS short-form Reactive-Proactive aggression scale	Those participting in arts had lower digital addiction and reactive aggression, and higher resilience, compared to newly-enrolled individuals.
CROSS-SECTIONAL + LONGITUDINAL STUDIES											
Bone et al. (2023)	USA	Nationally representative cohort		11,780 (50 % F)	11-21 (mean 15.02, SD 1.62) {Wave 1} 11-23 (mean 15.89, SD 1.64) {Wave 2}	Cross-sectional + Longitudinal	Participation in any of the following clubs: band; book club; chorus/choir; cheerleading/dance; drama club; newspaper; or orchestra (not separated)	Multiple/not separated	Loneliness Social support	“How often was the following true during the past week? You felt lonely.” Binary indicator created by collapsing response options into not lonely (never or rarely) versus lonely (sometimes, a lot of the time, most or all of the time). Asked how much they felt that their friends cared about them. Five response options ranged from not at all to very much. Binary indicator of peer social support was created by collapsing response options into low (friends care not at all, very little, somewhat) versus high (friends care quite a bit, very much).	Participating in extra-curricular clubs was associated with higher subsequent social support, even accounting for past social support. No significant associations with loneliness. Participating in more diverse activities associated with greater social support.
Yeo et al. (2023)	Singapore	Secondary schools in all parts of Singapore		1190 (57.6 % F)	12-19y	Cross-sectional + Longitudinal	Visual and Performing arts co-curicular activity groups	Multiple/not separated	Confidence	Four items, 7-point Likert scales	No significant differences in confidence for arts versus comparison group
COHORT/LONGITUDINAL STUDIES											
Bickham et al. (2015)	USA	Recruited from grades 7–9 in schools, after-school programs, and summer camps in a small city in the northeastern USA		126 (46.8 % F)	12.6–15.9 (mean 14.0) {baseline}	Cohort/longitudinal	Time spent using media category: 'music'	Music (listening)	Depression	Beck Depression Scale (minus suicidality component)	'Music' media use not longitudinally associated with depression (although is marginally associated contemporaneously)
Bone et al. (2022)	USA	Nationally representative cohort		AddHealth cohort: 10,106 (50 % F) NELS:88 cohort: 15,214 (50 % F)	AddHealth: mean 15.07 {Wave 1} NELS:88: ≤13y - 1 % 14y - 63 % 15y - 31 % ≥16y - 5 % {Wave 1}	Cohort/longitudinal	Participation in any of the following clubs: band/orchestra, chorus/choir, dance, drama club, and student newspaper. Parent report of if heir child attended classes outside school: art; music; dance; and the history and culture of their ancestors. Parents also asked if child: borrows books from the public library; attends concerts or other musical events; goes to art museums; goes to science museums; and goes to history museums. Latent construct of 'arts and culture' created from the above.	Multiple/not separated	Reportedly antisocial and criminal behaviour (RACB) Self-control	Self-report across multiple questions Self-reported list of questions (previously used in past research)	W1 engagement in clubs associated with reduced RACBs and increased self-control concurrently. Associated with reduced W2 RACBs. All other associations mediated through other waves' outcomes
Fluharty et al. (2023)	USA	Nationally representative cohort	5th and 8th graders	8315 (47.8 % F)	mean 11.2 (SE 0.01) {5th grade} mean 14.3 (SE = 0.01) {8th grade}	Cohort/longitudinal	Parent report of if child had participated in last year in: dance lessons, music lessons, art classes or lessons, organised performing art programmes. Teacher report of frequency that children had lessons or projects on: music, musical instruments, art, or art materials. School administrator report of adequacy of following school facilities: art room, music room, auditorium.	Multiple/not separated	Individual externalising behaviours School-level externalising behaviours	Parent-report Strengths and Difficulties Questionnaire (externalising subscale) Frequency of 6 externalising behaviours [class cutting, physical confrontations, theft, vandalism, bullying, and classroom disorder] occurring in the school, with responses on a five-point scale ranging from ‘never’, to 'occasionally', 'once a month', 'once a week', and ‘daily’. Responses were summed to create an externalising behaviours index.	Participation in higher number of different arts-based extra-curricular activities associated with reduced externalising behaviours at individual but not school level. Both results lower in magnitude when accounting for confounders
Foster and Jenkins (2017)	USA	Nationally representative cohort		2907 (49 % F)	5-18 {wave 2} 10-19 {Wave 3}	Cohort/longitudinal	Parent report of child taking lessons in any of the following: dance, drama, music-instrument, music-singing, music-not specified. Whether there is a musical instrument in the home and whether the child uses it.	Music (playing)	Behaviour problems Positive behaviours Global self-concept	Behaviour Problems Index Positive Behaviours Scale Ability self-concept scales	Positive associations between engagement and outcomes in raw comparisons and adjusted for confounders. No statistically significant differences in outcomes after adjusting for propensity to engage.
Huang et al. (2023)	China	Online sampled survey	Adolescents that use smartphones	2049 (51.8 % F)	mean 14.39 (SD 2.27)	Cohort/longitudinal	Frequency of listening to music on smartphone	Music (listening)	Depression Anxiety Somatization	CES-D-10 GAD 7 SSS-8	More music listening associated with better mental health, moreso than any other smartphone use content.
Kwak et al. (2018)	USA	National survey of target population	Involved in child welfare system	790 (58.1 % F)	12-16 (mean 15.8, SD 1.0) {Wave 1, follow up ∼36 months later}]	Cohort/longitudinal	Belonging to art and music clubs (Examples of art and music clubs included bands, choirs, dance clubs, and drama clubs)	Multiple/not separated	Delinquency Depressive symptoms Trauma symptoms	Modified Self-Report of Delinquency Scale CDI TSCC	Participation in arts/music clubs associated with increased trauma symptoms.
Kwon et al. (2020)	South Korea	Middle schools in Seoul		3633 (46.9 % F)	Middle school (12–14)	Cohort/longitudinal	Frequency of music concert attendance (both in school programs and individually)	Music (listening)	Subjective well-being (cognitive and affective aspects of life satisfaction and the experience of emotion)	Questions from survey (previously validated - negative wordings omitted) 6 included items scored on 5-point Likert-type scale ranging from 1 = not at all to 5 = very much	Music concert attendance associated with higher subsequent subjective wellbeing. Increase in SWB higher for those with worse initial SWB.
O'Flaherty et al. (2022)	Australia	Nationally representative cohort		3401 [5206 obs]	12-13y (mean 12.9) {T1} 14-15y (mean 14.9) {T2}	Cohort/longitudinal	Extra curricular activity engagement in the last week: Art, music, or performance lessons (e.g., piano, dance, choir, or drama)	Multiple/not separated	Psychosocial health: reactivity persistence introversion Depressive symptoms Externalising symptoms Internalising symptoms HRQoL: emotional, social	School-age Temperament Inventory SMFQ SDQ PedsQL	Small but significant relationships between extra-curricular arts participation and multiple outcomes, although only results for introversion significant in fixed-effects estimation.
PRE-POST COMPARISON STUDIES											
Felsman, 2019 (Ch.2)	USA	Middle and high schools in Detroit	Those screening positive for social phobia (some analyses)	147 (57 % F)	8th Grade - 12th Grade (13-18y)	Pre-post comparison	"The Improv Project” - 10 week improvisational theatre course	Drama	Social self-efficacy	MINI-SPIN	Program resulted in reduction in social anxiety for those that screened positive at T1 for social phobia, but not for the full sample. Changes correlated with changes in social skills, creative-self efficacy, and hope.
Felsman, 2019 (Ch.4)	USA	Middle and high schools in Detroit		339 (∼50 % F)	8th Grade - 12th Grade (13-18y)	Pre-post comparison	"The Improv Project” - 10 week improvisational theatre course	Drama	Social self-efficacy Social Anxiety Intolerance of Uncertainty	Adolescent Social Self-Efficacy Scale MINI-SPIN Brief Intolerance of Uncertainty Scale (Brief-IUS)	Program associated with significant reductions in intolerance of uncertainty and social anxiety, marginal increase in self-efficacy
McGrath and Stevens (2019)	Australia	South Australian Circus Centre's (SACC) Cirkidz Circus School in Adelaide, South Australia	'Tweenz' that partake in SACC programmes	23	8-14 (mean 10)	Pre-post comparison	20 weeks of circus arts training in classes provided by Cirkidz (for beginners, for fun)	Other (Circus Arts)	Psychological wellbeing Social Support and Peers	Kidscreen-27	No statistically significant changes to wellbeing and social support following the program.
Swami and Shaw (2019)	UK	One school in Essex		14 (78.6 % F)	16-18y (mean 16.8, SD 0.58)	Pre-post comparison	Extra-curricular life drawing classes	Arts and crafts	Self-esteem	Rosenberg	No significant difference in self-esteem before and after life drawing
QUASI-EXPERIMENTAL STUDIES											
Saramento et al. (2024)	Portugal	One public school In which the SEL initiative was run	Individuals that took part in the SEL initiative	116 (50 % F)	9th - 11th grade (15-18y, mean 15.2)	Quasi-experimental	Socio-emotional learning program involving the running of an online radio station (script-writing, planning, recording), as well as some well-being and citizenship sessions, over whole school year	Other (online radio)	Socio-emotional skills: Resilience (persistence) Adaptability optimism	Survey on Social and Emotional Skills (SSES) - both students and teachers	Increase in teacher-perceived socio-emotional skills from the program (but not student-perceived).
RCT STUDIES											
Han (2020)	China	Junior and high schools in Beijing		100	13-17 (mean 14.3)	RCT	Listening to 'lively music' or 'soothing music' at a fixed time each day for 6 months	Music (listening)	Depression	Beck Depression Inventory	Regular and long-term listening to music can reduce depressive symptoms. Brisk music may be more impactful than soothing music

#### 3.2.1 Study settings and design

Studies were conducted across a wide range of countries; the USA (n = 11), Australia (n = 3), China (n = 3), the UK (n = 3), Turkey (n = 1), Brazil (n = 1), Canada (n = 1), Czech Republic (n = 1), Portugal (n = 1), Singapore (n = 1), South Korea (n = 1), Spain (n = 1), and Thailand (n = 1).

Schools were the most common setting for studies (n = 12). Some studies were conducted in youth centres/extra-curricular education centres (n = 4). Many used existing nationally representative survey data (n = 11) with some collecting new data via online surveys (n = 2).

Cohort and longitudinal designs were most commonly used (n = 14), followed by cross-sectional designs (n = 13). One randomised control trial of a school-wide program was identified, and one quasi-experiment.

#### 3.2.2 Arts and creative activities

Studies investigated a variety of arts and creative activities. The highest number of results related to music (19/55 results), with nine discussing playing/performing music, and seven focusing on listening to music (one on live music). The other three included both or did not delineate. Drama was the next most commonly investigated (5/55), followed by visual arts/arts and crafts (4/55). Other activities were yearbook making (3/55), circus arts (2/55), and an online radio show (1/55). 20 results related to multiple categories of arts or did not distinguish between arts activities.

#### 3.2.3 Outcomes

Across the 28 included studies and 55 results, 49 mental health and wellbeing outcomes were investigated. Most studies (n = 17) only investigated one outcome. The highest number of mental health and wellbeing outcomes assessed in a single study was five (n = 1; O'Flaherty et al., 2022).

A plurality of included studies (19/55 results) investigated internalising mental health symptoms (such as symptoms or clinical diagnosis of anxiety or depression), followed closely by overall wellbeing or indicators of wellbeing (16/55 results). Other studies outcomes included externalising behaviours (such as behavioural problems, aggression, and delinquency), as well as addictive behaviours, and outcomes related to self-perception (e.g. self-esteem, self-efficacy, confidence).

#### 3.2.4 Study results

The majority of identified results showed positive associations between engaging in arts and creative activities and mental health (33/55). Only 4/55 results showed a negative association, and a further 3/55 showed mixed results (positive/negative for different groups). The remaining 15/55 results did not show statistically significant associations in either direction.

Directions of significant results broken down by arts activity category and outcome category are summarised in Appendix Figures A4-A16.

### 3.3 Critical appraisal

12/28 included studies (32/55 results) were rated as ‘strong’, 12/28 (19/55 results) were rated as ‘moderate’, and the remaining four (4/55 results) were rated as ‘weak’. Of the weak cross-sectional studies (n = 3), issues tended to be around poor explanation of sampling and inclusion, and a lack of identified or included confounding factors. The only RCT (Han, 2020) was rated ‘weak’, mainly due to unclear reporting of assignment and blinding. Where studies were rated moderate, the key issue was that no or minimal confounders were identified or adjusted for.

**Fig. 2:**
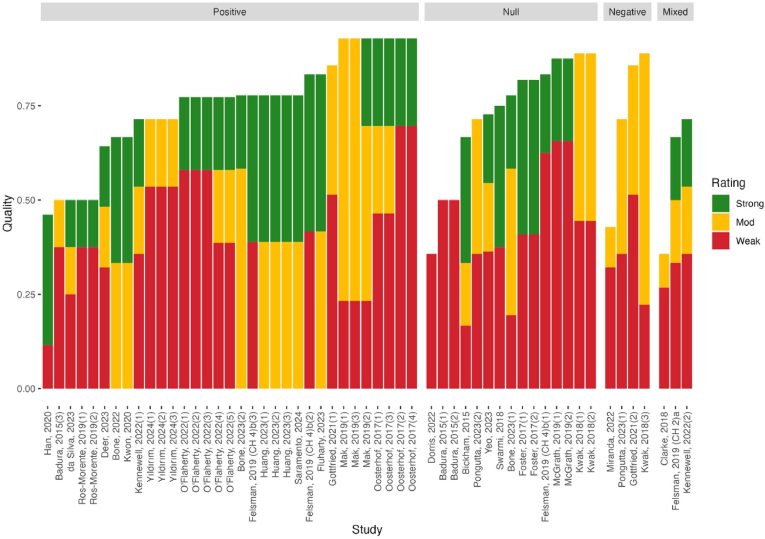
Harvest plot showing the direction, methodological quality, and support for BH criteria of the 55 results from included studies **Note:** Results are grouped by direction, with positive referring to a result which suggests that arts engagement is associated with improved mental health or reduced symptoms, and negative referring to the opposite. Bar height represents methodological quality, as appraised using the relevant JBI Critical Appraisal Tools (Quality = % of applicable criteria satisfied). Bar fill represents the proportion of the four result-level BH criteria (strength, temporality, dose-response, and experimental/quasi-experimental evidence) rated ‘strong’, ‘moderate’ or ‘weak’ for each result.

Fig. 2 presents the quality score (% of applicable criteria satisfied) for each study by the direction of the association (as well as Bradford Hill viewpoint support). Appendix Table A5 presents the scores for each result individually.

There was considerable heterogeneity in methodological quality across different arts and creative activities. Both arts and crafts and drama had no methodologically ‘weak’ results. Studies investigating music had the most weak results (3/19 results), but this may be by virtue of having the most studies of any specific arts category. Studies investigating multiple or non-specific arts activities had the highest number and proportion (12/21) of methodologically strong studies, as well as no weak studies.

The GRADE summary of findings table, rating the certainty of findings, is presented in Appendix 5.

### 3.4 Causal evaluation

**Fig. 3:**
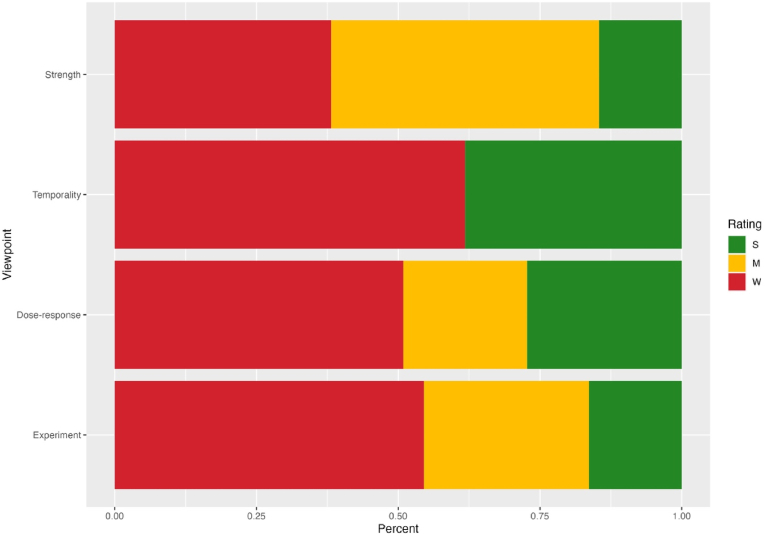
Proportions of results from included studies providing 'weak', 'moderate', and 'strong' support for each of the BH viewpoints **Note:** Coloured fill of bars represents the proportion of the 55 results from included studies that provide different levels of support for each BH viewpoint. S = Strong, M = Moderate, W = Weak.

We evaluated studies against the Bradford Hill viewpoints on causality (Hill, 1965). Fig. 3 presents the proportion of included studies that provide strong, moderate, or weak support for each BH viewpoint (at result level). Support for each viewpoint by individual result can be found in Appendix Table A5.

Fig. 2 presents a harvest plot showing the methodological quality and support for result-level BH viewpoints, grouped by direction of effect. Analogous plots broken down by outcome category and arts activity type can be found in Appendix 6.

#### 3.4.1 Strength of association

The majority of results (34/55) had moderate or strong support for this viewpoint, meaning that they reported statistically significant relationships between arts engagement and adolescent MHWB. Where results were deemed weak, this was generally because results were not statistically significant, or already small significant results did not hold up to changes in specification (such as adjusting for confounding). For example, O'Flaherty et al.’s (2022) results of small but significant relationships between extra-curricular arts engagement and MHWB outcomes became statistically insignificant once adjusted for individual-level time-invariant confounding.

Whilst we primarily considered statistical significance and robustness of results when assessing support for this viewpoint, it may also be important to consider the magnitude of significant associations. We did this by assessing standardised effect sizes against commonly used thresholds (see Appendix 2). Under these criteria, only 8/55 results were deemed to provide strong support for this viewpoint, meaning that they had ‘large’ effect sizes. However, it is notable that one of these strong associations was found only in a sub-population; Felsman (2019; Ch.2) found that a 10-week improvisational theatre course had a large impact (in terms of both regression coefficient and reduction in caseness) on social self-efficacy in those who initially screened positive for social phobia, but not in the full study population. When considering different arts activities, it is notable that music listening had a higher proportion of strong results in relation to this viewpoint compared to other activities (Appendix Figure A2).

When we consider only results rated methodologically moderate or strong according to our quality appraisal criteria, support for this viewpoint remains relatively similar; 7/51 results were strong for strength of association, and 33/51 were moderate. Under our criteria for this viewpoint, the body of evidence seems to provide moderate support. However, this may not necessarily harm the case for causality, and we return to this in the discussion.

#### 3.4.2 Temporality

Temporality is often considered a pre-requisite for causal conclusions and is generally the least contentious of the BH viewpoints. 21/55 results were rated strong for this viewpoint, as the measure of the exposure pre-dated the measure of the outcome. Weak studies (34/55 results) were rated so due to cross-sectional designs, which cannot rule out reverse-causality. It is notable that all of the studies investigating drama activities were rated strong for this viewpoint (Appendix Figure A1), and those looking at addictive behaviour outcomes were all rated weak (Appendix Figure A3), although these both had low numbers of studies (four and three respectively).

3/4 negative results came from studies rated weak for this viewpoint, which may suggest that these negative results may be the result of confounding or reverse-causality. 11/21 results rated strong on this viewpoint were positive. If we again remove those results rated methodologically weak, we find that the proportion of strong results for this viewpoint increases slightly (20/51). Therefore, it seems that the evidence provides moderate support for the temporality viewpoint, and further studies that are temporally correct (i.e. the exposure is measured before the outcome) may be necessary to draw stronger conclusions.

#### 3.4.3 Dose-response

Most studies were considered weak in terms of dose-response (28/55), as they used a binary measure of engagement with arts and creative activities. 12/55 results were rated moderate, including at least an ordinal or continuous measure of exposure. 15/55 results were considered strong, using both continuous measures of exposure and outcome. Studies investigating music listening had the strongest support for this outcome (Appendix Figure A3), whereas there was no evidence to support a dose-response relationship with regards to drama activities (Appendix Figure A2). For example, Huang et al. (2023) found that increased music listening frequency was associated with improvements in multiple domains of mental health (using validated continuous scales), and Oosterhoff et al. (2017) found that having spent more years engaging with music clubs in school was associated with a lower frequency of illicit substance use.

A number of different ways of characterising dose-response were identified. Five results considered breadth of engagement (e.g. number of different activities), seven results considered frequency of engagement (e.g. number of times a year an activity is done), seven results considered intensity of engagement (e.g. time spent doing an activity), and eight results considered persistence of engagement (e.g. over how many years a young person had done an activity). Of these, persistence had the highest number of strong methodological quality (7/8 results), and intensity had the fewest (0/7). It is also notable that studies investigating frequency of engagement found exclusively positive results.

Dose-response had the second highest proportion of weak support of all of the viewpoints (Fig. 2). It is notable, though, that of all results considered strong in this category, almost all (12/15) were significant positive associations, with two mixed (Kennewell et al., 2022) and one null result (Bickham et al., 2015). Furthermore, upon removing methodologically weak studies, again this increases the number of results providing moderate or strong support for this viewpoint; 15/51 provide string support, and 11/51 moderate. Therefore, despite the low number of studies that consider a dose-response, those that do could be considered to provide moderate support for this viewpoint.

#### 3.4.4 Experimental/quasi-experimental evidence

A majority of results were considered weak in terms of experimental/quasi-experimental evidence (30/55). This was determined by study design, with these studies all being cross-sectional or uncontrolled pre-post analyses. 16/55 results came from studies judged to have moderate experimental evidence, which were generally longitudinal studies. Only 9/55 results were judged as strong for this viewpoint, which included RCTs but also quasi-experimental and statistical methods such as fixed effects estimation and propensity score matching (Craig et al., 2025). We note here that different statistical and quasi-experimental techniques require different assumptions for results to suggest causal conclusions, and their use does not automatically guarantee causality. As such, we do not consider these methods to necessarily provide causal conclusions, but consider only that (if well-implemented) they can reduce confounding and mimic some conditions of RCTs (Craig et al., 2012). Experimental/quasi-experimental evidence was entirely moderate or strong for studies on music listening, but was lacking for music playing (Appendix Figure A2).

20/55 results were also found in studies which included some prior measure of MHWB. Whilst this is not included as part of our definition of experimental/quasi-experimental evidence, it is particularly key in examining the relationship between arts and MHWB, due to the likely presence of reverse-causality (Bone & Fancourt, 2022).

Experimental/quasi-experimental evidence had the smallest proportion of strong support of all viewpoints (Fig. A2). However, it is notable here that all but one of the negative results found in this review came from studies judged weak on this viewpoint, which once again may suggest that negative results are due to selection or confounding on some level. When we again remove methodologically weak studies, this increases the proportion of studies providing moderate support for this viewpoint but not strong; 16/51 were rated moderate, and 8/51 strong. The body of evidence therefore provides weak to moderate support for the experimental/quasi-experimental evidence viewpoint. It does appear to be lacking in support in comparison with other viewpoints, and considerably more evidence is needed which can better account for confounding.

#### 3.4.5 Plausibility/coherence

Multiple included studies posited mechanisms through which regular engagement may affect adolescent mental health and wellbeing. For example, Bone et al. (2022) found self-control to be a significant mediator in their models linking arts and cultural engagement to subsequent anti-social behaviour. Deer et al. (2023) found significant moderation via cognition and emotion when investigating the relationship between arts and cultural participation and self-efficacy.

In terms of the wider literature, theory and evidence has suggested many mechanisms through which arts and creative activities would plausibly improve mental health. In particular, a systematic review by Williams et al. (2023) found, particularly from qualitative interview-based studies of participatory arts programs, that there is good evidence of therapeutic processes. These include creating a protected space away from everyday life, and fostering connection and team spirit. In analysis by Fancourt et al. (2023), mechanisms such as the improvement of self-control and emotional regulation were highlighted and empirically tested. Furthermore, imaging studies have demonstrated neurobiological changes in response to arts (Sharma & Silbersweig, 2018). Systematic reviews have found that engaging in creative tasks increases alpha and theta waves in the brain associated with relaxed states, meditation, and memory retrieval (Pidgeon et al., 2016), as well as activating the neural circuits associated with emotional regulation (Barnett & Vasiu, 2024). However, the vast majority of this research is in adults, and much of it focuses on art therapies. Therefore, our identified body of evidence, placed within the broader context, provides moderate to strong support for viewpoint of plausibility, but more research is needed into the specific mechanisms of action of regular arts engagement on adolescent MHWB.

#### 3.4.6 Consistency

More than half of identified results were positive and statistically significant (33/55). These span a range of countries, settings, arts activities, outcomes, and methodologies. Out of the identified negative results (4/55), one of these was a study conducted on a specific subset of the population of interest (children involved in the welfare system; Kwak et al., 2018). Another was considered low quality, and was judged as weak on the majority of BH viewpoints (Miranda, 2022). Studies with mixed results (3/55 results) also tended to focus on specific subsamples (e.g. Clarke & Basilio, 2018). The remaining results (15/55) were not statistically significant. If we were to exclude results from studies of low methodological quality, the proportion of significant, positive results increases (31/51), and the proportion of negative (3/51) and mixed (2/51) decrease. The proportion of null results is essentially unchanged (14/51).

One concern for consistency may be publication bias, although the inclusion of unpublished work (pre-prints, working papers, doctoral dissertations, and grey literature), as well as the number of null results identified, mitigate this. Furthermore, it is difficult to draw concrete conclusions on the consistency of this body of evidence due to the vast heterogeneity of included arts and creative activities, as well as outcomes of interest. However, the fairly consistent direction of effects across such heterogenous activities and settings, this provides at least some support for the viewpoint of consistency when broadly considering the relationship between arts engagement and adolescent MHWB.

## 4 Discussion and conclusions

### 4.1 Discussion

We systematically reviewed the published literature linking regular engagement with arts and creative activities and MHWB in adolescents. We found that most of the evidence demonstrated a consistent positive relationship, which concurs with previous summaries of the literature (Bone & Fancourt, 2022; Fancourt et al., 2023). There were many studies finding a positive relationship between music (especially music listening) and adolescent MHWB. In terms of outcomes, there was a large amount of evidence linking arts engagement with better wellbeing, as well as with reduced internalising symptoms.

We then evaluated the extent to which this evidence base supports causal conclusions using the Bradford Hill viewpoints (Bradford Hill, 1965). The evidence provides at least some support for all viewpoints.

Moderate support for temporality is reassuring, as it mitigates the potential for results to be driven by reverse-causality, which is very plausible in the relationship between arts and mental health. Therefore, moderate to strong support for temporality (although not sufficient) across this body of evidence is important as a prerequisite for a causal relationship. Studies using more advanced methods should continue to ensure that they are temporally correct (i.e. the outcome is measured after the exposure), in order to be consistent with a causal effect.

Weak to moderate support was found regarding strength of association. However, this viewpoint has been applied in a variety of ways in other causal reviews (Shimonovich et al., 2022), and if we were to focus entirely on statistically significant results rather than magnitude (as suggested by Fedak et al., 2015), a much higher proportion of results would have been considered ‘strong’. This may be pertinent as we may not expect to find ‘large’ effects in psychology and psychiatry research, and ‘small’ changes in MHWB indicators may lead to large impacts (Carey et al., 2023). Indeed, Bradford Hill himself advises not to dismiss a causal hypothesis due to small in magnitude associations (Hill, 1965).

Dose-response had fairly weak support. However, Hill (1965) suggests that the existence of a dose-response only bolsters the case for causality, and having found predominantly binary relationships does not hamper causal interpretations. Binary associations may suggest that engaging with *any* arts is better for adolescent MHWB than none. We may not necessarily expect to see a monotonic increasing dose-response to arts and creative engagement; the over-scheduling hypothesis suggests that young people who spend a large portion of their time participating in organised out-of-school activities may be at-risk for poor developmental outcomes. However, evidence of this in adolescence is mixed (Mahoney & Vest, 2012). Furthermore, the dose-response evidence found in this review spans breadth, frequency, intensity, and persistence of engagement. Evidence investigating the existence of a dose-response may, though, be important not only for bolstering the case for a causal relationship, but it also may be relevant to decision-makers; whether such a relationship exists indicates whether it would be effective to try and increase the *level* of arts engagement in young people to improve mental health, or if just encouraging young people to engage in *any* arts at all would be enough.

Experimental/quasi-experimental evidence also had weak support, and a distinct lack of results constituting ‘strong’ evidence. We used broad criteria for what constituted experimental and quasi-experimental evidence, including statistical techniques that attempt to mimic experimental conditions by reducing confounding. This was in part due to the low number of RCT-design studies, perhaps due to the issues that arise when trying to conduct RCTs with adolescents and young people (Nevill, 2019). Despite these broad criteria, we still found weak support, which suggests that more needs to be done to apply methods that reduce the impact of confounding and potential selection into arts activities based on personal characteristics that may also impact mental health. These findings more formally echo the suggestion by Bone and Fancourt (2022) that stronger statistical methods are needed in research on arts engagement and health to better deal with the problems of confounding, selection, and reverse-causality that regularly arise. A wide array of quasi-experimental methods exist that can reduce confounding and allow for strong causal inference without RCTs (Bärnighausen et al., 2017; Craig et al., 2012), provided that identifying assumptions are met. Studies should further apply these kinds of methods to strengthen this evidence base in a way that can move beyond observation and correlation, although it is key that methods are applied appropriately to available data, and that choices are informed by assumptions and theory around the nature of underlying processes. For example, regression adjustment and propensity score methods require the assumption that all potential sources of confounding are included in the model (no unobserved confounding) for estimates to be considered causal, and fixed effects estimation requires that all time-varying sources of confounding are adjusted for (Wooldridge, 2010). Without careful and thorough application, statistical methods and the relationships they find cannot be considered causal, and may risk being misleading if improperly used.

There are many plausible mechanisms of action through which arts engagement may affect MHWB, and the existence of such a causal effect would not conflict with existing understanding. However, more exploration of the specific mechanisms of *regular* engagement on *adolescent* MHWB may be warranted.

### 4.2 Strengths and limitations

To the best of our knowledge, this is the first systematic review to examine the relationship between regular engagement with arts and creative activities and adolescent mental health and wellbeing. It expanded upon recent reports (Bone & Fancourt, 2022; Fancourt et al., 2023) and related but different past reviews (Daykin et al., 2008; Viola et al., 2024; Zarobe & Bungay, 2017). We applied the Bradford Hill viewpoints as a means to better understand the extent to which the evidence base can support causal interpretations, which has not been done before in the field of arts and health.

A key strength of this review is its scope; we included studies from around the world on a wide variety of arts and creative activities and across a wide range of mental health and wellbeing outcomes. However, this also results in the limitation that we were unable to undertake any quantitative synthesis of the evidence, such as meta-analysis, due to the heterogeneity of included studies and results. Further reviews could have a tighter scope, and focus on specific activities or outcomes in order to allow for this more granular quantitative synthesis.

We focused on specifically adolescence (10-19y) with regards to both exposure and outcome, as this is a key group in which mental health trends are most concerning, and also as focusing on secondary school-aged young people allows for a clear opportunity for intervention to increase arts engagement (in school). There are likely also positive benefits of arts engagement in adolescence that manifest in adulthood, and these should also be explored, but this was outside the scope of this study.

A large portion of screening at both title/abstract and full-text stages was undertaken by one reviewer. However, at each stage a sample of records were dual-screened and discussed in order to maximise consistency and minimise bias. We also undertook pilot testing of screening tools to ensure clarity.

### 4.3 Conclusions

There is a large and growing body of evidence supporting a positive association between regular engagement in arts and creative activities and adolescent MHWB. This is consistent across a wide range of settings, creative activities, and outcome measures.

The body of evidence provides moderate support for causal conclusions, but some areas lack evidence more than others. In particular, more experimental evidence is also needed to bolster causal conclusions. Researchers should seek to implement experimental and quasi-experimental statistical methods that can account for confounding from reverse causality and bi-directionality, as well as selection and unobserved heterogeneity. Alongside traditional RCTs, this may include instrumental variable or regression discontinuity designs (Craig et al., 2012), as well as individual fixed effects within-person designs (Bärnighausen et al., 2017). Future research should also seek to evaluate whether there is a dose-response relationship.

Despite these gaps, the evidence clearly suggests that strategies to increase young people's access to and engagement with arts and creative activities may be an effective avenue through which to protect and improve their MHWB. Given the existing inequalities in access to and engagement with arts (Mak & Fancourt, 2021; Rodriguez et al., 2024), it is important to consider how these benefits can be equitably distributed, through, for example, subsidised provision (Evans, 2016; O'Hagan, 2024).\

## CRediT authorship contribution statement

**Sam Hugh-Jones:** Writing – review & editing, Writing – original draft, Visualization, Project administration, Formal analysis, Data curation, Conceptualization. **Stephanie Ray:** Writing – review & editing, Formal analysis, Data curation. **Anna Wilding:** Writing – review & editing, Supervision, Conceptualization. **Matt Sutton:** Writing – review & editing, Supervision, Conceptualization. **Neil Humphrey:** Writing – review & editing, Supervision, Conceptualization. **Luke Munford:** Writing – review & editing, Supervision, Conceptualization.

## Competing interests

The authors have no competing interests to declare.

## Ethics

Ethical approval was not required for this study.

## Source of funding

SHJ is funded by a 10.13039/501100000265Medical Research Council Doctoral studentship.

## Declaration of competing interest

The authors declare that they have no known competing financial interests or personal relationships that could have appeared to influence the work reported in this paper.

## Data Availability

No data was used for the research described in the article.

## Footnotes

1 E.g. where individuals are asked “How often do you do *X*?”

2 Had agreement been less than ‘substantial’ (k < 0.61), inclusion criteria would have been re-visited and records would have been re-screened following discussion.

3 Temporality was rated as only ‘strong’ or ‘weak’.
