# Supplementary material for: Does regular engagement with arts and creative activities improve adolescent mental health and wellbeing? A systematic review and assessment of causality
Source: SSM Popul Health. 2025 Jul 22;31:101845. doi: 10.1016/j.ssmph.2025.101845 (PMC12319337; doi:10.1016/j.ssmph.2025.101845)
Supplement: Multimedia component 1 [file mmc1.docx]

**Does regular engagement with arts and creative activities improve adolescent mental health and wellbeing? A systematic review and assessment of causality**

Appendices

Appendix 1. PRISMA-2020 Checklist

Table A1

| **Section and Topic** | **Item #** | **Checklist item** | **Notes** | **Location where item is reported** |
| --- | --- | --- | --- | --- |
| **TITLE** | | |  |  |
| Title | 1 | Identify the report as a systematic review. |  | Title |
| **ABSTRACT** | | |  |  |
| Abstract | 2 | See the PRISMA 2020 for Abstracts checklist. |  | Abstract |
| **INTRODUCTION** | | |  |  |
| Rationale | 3 | Describe the rationale for the review in the context of existing knowledge. |  | Section 1 |
| Objectives | 4 | Provide an explicit statement of the objective(s) or question(s) the review addresses. |  | Section 1.4 |
| **METHODS** | | |  |  |
| Eligibility criteria | 5 | Specify the inclusion and exclusion criteria for the review and how studies were grouped for the syntheses. |  | Section 2.2 |
| Information sources | 6 | Specify all databases, registers, websites, organisations, reference lists and other sources searched or consulted to identify studies. Specify the date when each source was last searched or consulted. |  | Section 2.1 |
| Search strategy | 7 | Present the full search strategies for all databases, registers and websites, including any filters and limits used. |  | Appendix 4, Table A3 |
| Selection process | 8 | Specify the methods used to decide whether a study met the inclusion criteria of the review, including how many reviewers screened each record and each report retrieved, whether they worked independently, and if applicable, details of automation tools used in the process. |  | Section 2.2-2.3 |
| Data collection process | 9 | Specify the methods used to collect data from reports, including how many reviewers collected data from each report, whether they worked independently, any processes for obtaining or confirming data from study investigators, and if applicable, details of automation tools used in the process. |  | Protocol: CRD42024610518 |
| Data items | 10a | List and define all outcomes for which data were sought. Specify whether all results that were compatible with each outcome domain in each study were sought (e.g. for all measures, time points, analyses), and if not, the methods used to decide which results to collect. |  | Protocol: CRD42024610518 |
|  | 10b | List and define all other variables for which data were sought (e.g. participant and intervention characteristics, funding sources). Describe any assumptions made about any missing or unclear information. |  | Protocol: CRD42024610518 |
| Study risk of bias assessment | 11 | Specify the methods used to assess risk of bias in the included studies, including details of the tool(s) used, how many reviewers assessed each study and whether they worked independently, and if applicable, details of automation tools used in the process. |  | Section 2.4 |
| Effect measures | 12 | Specify for each outcome the effect measure(s) (e.g. risk ratio, mean difference) used in the synthesis or presentation of results. | Given heterogeneity of results, only directions of effects were presented | N/A |
| Synthesis methods | 13a | Describe the processes used to decide which studies were eligible for each synthesis (e.g. tabulating the study intervention characteristics and comparing against the planned groups for each synthesis (item #5)). | All studies were eligible for synthesis | N/A |
|  | 13b | Describe any methods required to prepare the data for presentation or synthesis, such as handling of missing summary statistics, or data conversions. |  | Section 3.4 |
|  | 13c | Describe any methods used to tabulate or visually display results of individual studies and syntheses. |  | Section 3.4 |
|  | 13d | Describe any methods used to synthesize results and provide a rationale for the choice(s). If meta-analysis was performed, describe the model(s), method(s) to identify the presence and extent of statistical heterogeneity, and software package(s) used. | No meta-analysis was undertaken | N/A |
|  | 13e | Describe any methods used to explore possible causes of heterogeneity among study results (e.g. subgroup analysis, meta-regression). | “ | N/A |
|  | 13f | Describe any sensitivity analyses conducted to assess robustness of the synthesized results. | “ | N/A |
| Reporting bias assessment | 14 | Describe any methods used to assess risk of bias due to missing results in a synthesis (arising from reporting biases). |  | Section 2.4 |
| Certainty assessment | 15 | Describe any methods used to assess certainty (or confidence) in the body of evidence for an outcome. |  | Section 2.4 |
| **RESULTS** | | |  |  |
| Study selection | 16a | Describe the results of the search and selection process, from the number of records identified in the search to the number of studies included in the review, ideally using a flow diagram. |  | Section 3.1 |
|  | 16b | Cite studies that might appear to meet the inclusion criteria, but which were excluded, and explain why they were excluded. | Too many studies were excluded for this to be feasible. Instead, common reasons for exclusion were listed in section 3.1 | N/A |
| Study characteristics | 17 | Cite each included study and present its characteristics. |  | Section 3.2, Table 5 |
| Risk of bias in studies | 18 | Present assessments of risk of bias for each included study. |  | Appendix 5 |
| Results of individual studies | 19 | For all outcomes, present, for each study: (a) summary statistics for each group (where appropriate) and (b) an effect estimate and its precision (e.g. confidence/credible interval), ideally using structured tables or plots. | No quantitative synthesis undertaken | N/A |
| Results of syntheses | 20a | For each synthesis, briefly summarise the characteristics and risk of bias among contributing studies. | No quantitative synthesis undertaken | N/A |
|  | 20b | Present results of all statistical syntheses conducted. If meta-analysis was done, present for each the summary estimate and its precision (e.g. confidence/credible interval) and measures of statistical heterogeneity. If comparing groups, describe the direction of the effect. | No quantitative synthesis undertaken | N/A |
|  | 20c | Present results of all investigations of possible causes of heterogeneity among study results. | No quantitative synthesis undertaken | N/A |
|  | 20d | Present results of all sensitivity analyses conducted to assess the robustness of the synthesized results. | No quantitative synthesis undertaken | N/A |
| Reporting biases | 21 | Present assessments of risk of bias due to missing results (arising from reporting biases) for each synthesis assessed. | No quantitative synthesis undertaken | N/A |
| Certainty of evidence | 22 | Present assessments of certainty (or confidence) in the body of evidence for each outcome assessed. | No quantitative synthesis undertaken | N/A |
| **DISCUSSION** | | |  |  |
| Discussion | 23a | Provide a general interpretation of the results in the context of other evidence. |  | Sections 3 + 4 |
|  | 23b | Discuss any limitations of the evidence included in the review. |  | Sections 3 + 4 |
|  | 23c | Discuss any limitations of the review processes used. |  | Section 4 |
|  | 23d | Discuss implications of the results for practice, policy, and future research. |  | Section 4 |
| **OTHER INFORMATION** | | |  |  |
| Registration and protocol | 24a | Provide registration information for the review, including register name and registration number, or state that the review was not registered. |  | Section 2 |
|  | 24b | Indicate where the review protocol can be accessed, or state that a protocol was not prepared. |  | Section 2 |
|  | 24c | Describe and explain any amendments to information provided at registration or in the protocol. |  | Appendix 3 |
| Support | 25 | Describe sources of financial or non-financial support for the review, and the role of the funders or sponsors in the review. |  | Title page |
| Competing interests | 26 | Declare any competing interests of review authors. |  | Title page |
| Availability of data, code and other materials | 27 | Report which of the following are publicly available and where they can be found: template data collection forms; data extracted from included studies; data used for all analyses; analytic code; any other materials used in the review. |  | Table 5 and Appendices |

*From:*  Page MJ, McKenzie JE, Bossuyt PM, Boutron I, Hoffmann TC, Mulrow CD, et al. The PRISMA 2020 statement: an updated guideline for reporting systematic reviews. BMJ 2021;372:n71. doi: 10.1136/bmj.n71. This work is licensed under CC BY 4.0. To view a copy of this license, visit <https://creativecommons.org/licenses/by/4.0/>

**Appendix 2. Applications of Bradford Hill criteria in this review**

Although the Bradford Hill (BH; Hill, 1965) criteria have been applied to assess causality in many reviews, there are not formal codified applications of each viewpoint. Therefore, the way in which each viewpoint was operationalised and evaluated in this review was informed by past causal reviews, and the context of this body of evidence. Shimonovich et al. (2022) noted that past reviews lacked consistency and transparency of application, so we provide this additional explanation here to aid understanding of our approach.

Some previous reviews applied various viewpoints at the point of searching and inclusion (i.e. systematic reviews of ‘causal’ studies). For example, Norman et al. (2016) incorporate both the temporality and experimental evidence viewpoints into their inclusion criteria. We chose, however, to evaluate viewpoints *after* searching and screening, as our aim was to understand what associations exist *and* the extent to which there is causal support for them. Causality is not considered a marker of quality in our review (hence why critical appraisal was undertaken separately).

Table A2 presents the ways in which each viewpoint was applied in this review, the level at which the viewpoint was applied, and the criteria used for the different levels of support. We chose to consider support at result level on an ordinal scale (‘weak’, ‘moderate’, or ‘strong’), in order to allow for comparability, yet provide more nuance than a binary classification. This is similar to many past causal reviews (Shimonovich et al., 2022). Viewpoints examined at the body-of-evidence level were evaluated qualitatively. Further explanation of the reasoning behind the characterisation of each of these viewpoints is provided below.

We chose not to apply the viewpoints of specificity and analogy. It seems unlikely that a specific relationship would be observed in the case of our question; there are many factors that can affect MHWB (Sawyer and Patton, 2018), and arts engagement may impact many outcomes other than just MHWB (Karkou et al., 2022). This has often been done in past causal reviews (Mente et al., 2009; Fedak et al., 2015; Norman et al., 2016), especially where mental health is an outcome (Moore et al, 2017). Analogy is one of the least-used of the BH viewpoints (Shimonovich et al., 2022), in part due to its nature as a highly subjective judgement (Stewart, 2020), which is “largely driven by the creativity of the investigators” (Mente et al., 2009, p.661). Indeed, Bradford Hill himself describes it as the weakest form of evidence. Therefore, we also chose not to evaluate this viewpoint.

**Strength of association**

Hill (1965) originally characterised this viewpoint as being about the *magnitude* of association. However, in an era with huge amounts of data, it has been argued that statistical significance is a more reasonable characterisation of a ‘strong’ association (Fedak et al, 2015). It is for this reason that many causal reviews focus on statistical significance over magnitude (e.g. Norman et al., 2016; Boniface et al., 2017; Moore et al., 2017). It is also the case that when considering social and environmental determinants of mental health, we may not expect to find large effects at all, but that does not negate the plausibility or importance of findings (Carey et al., 2023). We therefore focused our evaluation of this viewpoint on statistical significance. Bradford Hill (1965) himself advised not to dismiss a causal hypothesis due to small in magnitude associations. We do though, consider magnitude by examining effect sizes, and classifying moderate or large effect sizes which are statistically significant as ‘strong’. Classifications of effect sizes for different metrics can be found below in Table A2. Very few results were on the borderlines of these thresholds, meaning that our conclusions are not overly sensitive to these choices.

Table A2. Thresholds for ‘small’. ‘medium’ and ‘large’ effect sizes

| **Index** | **Thresholds** | **Reference** |
| --- | --- | --- |
| Cohen’s d | Small ≥0.2 Medium ≥0.5 Large ≥0.8 | Sullivan and Feinn, 2012 |
| Odds Ratio | Small 0.5<x<2 Medium ≥2, ≤0.5 Large ≥3, ≤0.33 | Sullivan and Feinn, 2012 |
| Correlation coefficient (Pearson / Spearmans) | Small 0 < \|x\| ≤0.3 Medium 0.3 < \|x\| ≤0.6 Large 0.6 < \|x\| ≤1 | Akoglu, 2018 |
| Standardised beta | Small 0.1 < \|b\| ≤0.29 Medium 0.29 < \|b\| ≤0.49 Large 0.49 < \|b\| | Cohen, 1988 |

**Temporality**

This is often considered the least contentious of the BH viewpoints (Stewart, 2020). We operationalise this similarly to other causal reviews, rewarding studies that are ‘temporally correct’ (i.e. the exposure measure pre-dates the outcome measure; e.g. Pickett and Wilkinson, 2015; Norman et al., 2016; Boniface et al., 2017; Livesy et al., 2019).

**Dose-response (Biological gradient)**

This is generally operationalised by rewarding studies which demonstrate some link between an increase in exposure and an increase (or decrease) in outcome (e.g. Stahl et al., 2013; Wilkinson and Pickett, 2015; Boniface et al., 2017). In terms of engagement with arts and creative activities, it is not always clear what should be considered the ‘dose’, so we rewarded studies with any exposure measure increasing in frequency, intensity, or breadth of engagement. Studies were further recognised for *also* using a continuous/ordinal outcome variable, as that allows for a greater understanding of potential dose-response relationships.

We recognise that this penalises studies investigating the extensive margin of arts engagement (i.e. *any* engagement versus *no* engagement), where these studies may still be of value, particularly with regards to policy-relevance. Causal relationships need not have a direct dose-response relationship, and more complex relationships can exist (Fedak et al., 2015). A lack of dose-response evidence would therefore not hamper the case for causality, but the existence would bolster it.

**Experimental / quasi-experimental evidence**

Experimental evidence is often considered the strongest form of evidence, and whilst Bradford Hill (1965) originally referred to “experimental, or semi-experimental evidence” (p.298), this has often been operationalised in causal reviews in relation only to randomised control trials (RCTs) and intervention studies (e.g. Mente et al., 2009; Stahl et al., 2013; Livesey et al., 2019). Given the expected lack of RCTs in relation to our study question, we chose to expand this to include natural and quasi-experimental methods (in which there is variation in ‘treatment’ that is out of the control of the researcher, but that need not be truly random; Craig et al., 2025) and causal inference methods which (under certain assumptions) can reduce confounding and mimic some conditions of RCTs (Craig et al., 2025).

Specifically, we applied the JBI ‘levels of evidence’ (Munn et al., 2014), which still gives RCTs pride of place, but also acknowledges the usefulness of quasi-experimental and other study designs (which we defined according to Craig et al. {2012}, Bärnighausen et al. {2017}, and de Vocht et al. {2021}).

**Plausibility / coherence**

Given that the difference between these two viewpoints is often unclear, we chose to evaluate them together (e.g. Stahl et al., 2013; Norman et al., 2016). Many studies evaluate plausibility based on evidence for ‘credible’ mechanisms (e.g. Stahl et al., 2013; Livesy et al., 2019). However, it is often unclear what constitutes a ‘plausible’ or ‘credible’ mechanism. Therefore, we choose not to make judgements about the quality of a suggested mechanism, and instead only take into account the existence of potential mechanisms of action within the identified body of evidence and the wider literature.

**Consistency**

It is generally agreed that this viewpoint refers to whether results appear consistent across study settings, populations, and research designs, meaning that by construction it is usually evaluated across the whole body of evidence. This is sometimes defined based on specific thresholds of proportions of studies that should have significant results in the same direction (e.g. Mente et al., 2009; Roffey et al., 2010). We instead chose to evaluate this viewpoint qualitatively, to avoid imposing an arbitrary cutoff.

Table A3. Applications of Bradford Hill (1965) viewpoints in this review

| **Viewpoint** | **Definition in this review** | **Level applied at (Result or body of evidence)** | **Assessment of support** |
| --- | --- | --- | --- |
| Strength | Statistically significant association, followed by magnitude of association | Result | **Weak** – no statistically significant relationships, or statistically significant relationships do not hold up to changes in specification / robustness checks  **Moderate** – At least one statistically significant relationship  **Strong** - Statistically significant relationship, with effect sizes considered ‘moderate’ or ‘strong’ (see Table A2) |
| Temporality | Is temporal order accounted for? I.e. are measures of arts engagement taken from before outcome measures? | Result | **Weak** - cross-sectional evidence, exposure and outcome are concurrent or outcome is measured before exposure  **Strong** - Exposure is measured before outcome |
| Dose-response (Biological Gradient) | Is there evidence that more/less exposure is associated with better/worse mental health? | Result | **Weak** – Binary measure of exposure  **Moderate** - Some variation in exposure in either frequency, intensity, or breadth (such as ordinal categories), outcome may still be binary  **Strong** - Continuous/ordinal exposure *and* outcome |
| Experimental / Quasi-experimental Evidence | Do findings come from experimental evidence? If not, do studies use methods to account for quasi-experimental methods, or at least observational designs which are adjusted for confounding | Result | [based on JBI ‘levels of evidence’ (Munn et al., 2014), with ‘quasi-experimental’ designs defined as in MRC guidelines (Craig et al., 2012)]  **Weak** – JBI Level 4 or below, pure observational  **Moderate** – JBI Level 3 (redefined as longitudinal/cohort studies)  **Strong** - JBI Levels 1-2 (RCTs + natural experimental methods defined by MRC – fixed effects, matching, difference-in-difference etc.) |
| Plausibility / Coherence | Are there credible mechanisms for effect? Is there any evidence for these mechanisms? | BoE | Evaluated qualitatively |
| Consistency | Significant associations with consistent directions found across settings, populations, methodologies | BoE | Evaluated qualitatively |

**Appendix 3. Deviations from published protocol**

There were no major deviations from the published protocol. Some minor deviations were made:

- The definition of ‘regular’ arts in the inclusion criteria was tightened to refer to specifically to arts “on more than one distinct occasion over the course of more than one week”, following peer review comments. Given that this represents exclusively a tightening of criteria, it should only have affected studies that were already included, however we still returned to and re-screened these under the new criteria. No new studies were identified for inclusion. During this process, we also assessed whether changing this criteria to a different number of discrete events or weeks would affect the inclusion of any previously included studies, which it did not.
- We were unable to search the Culture, Health and Wellbeing database as access to the database was unavailable over the timeline of this project.
- Given the heterogeneity of outcome variables included, it was decided not to undertake GRADE at the outcome level. Instead, GRADE was applied to the entire body of evidence, considering the entire span of ‘mental health and wellbeing’ as the outcome of interest. This was not considered an issue, as the quality appraisal and causal evaluation cover many aspects of certainty of evidence. It is also for this reason that publication bias was not undertaken quantitatively.

**Appendix 4. Example search strategy**

Table A4.

| **Concept** | **Search no.** | **Query** |
| --- | --- | --- |
| Arts and creative activities | 1 | ((Art or Arts or "Participatory art*" or "Art* participat*" or "Art* activit*OR Art* engag*" or Music* or Sing* or song* or Drama or theat* or Danc* or movement or Paint* or draw* or collage or "arts and crafts" or sculpt* or pottery or Poetry or poem* or storytelling or "creative writing").ab. or (Art or Arts or "Participatory art*" or "Art* participat*" or "Art* activit*OR Art* engag*" or Music* or Sing* or song* or Drama or theat* or Danc* or movement or Paint* or draw* or collage or "arts and crafts" or sculpt* or pottery or Poetry or poem* or storytelling or "creative writing").ti.) not therap*.af. not "operating theat*".af. |
| Adolescence | 2 | (Adolescen* or Teen* or Youth or "young person" or "young people" or "young adult*").ab. or (Adolescen* or Teen* or Youth or "young person" or "young people" or "young adult*").ti. |
| Mental health / wellbeing | 3 | (((mental or emotional or psychological or psychiatric or internalising or internalizing or anxiety or depressive) and (health or illness* or disorder* or symptom* or distress or difficult*)) or Wellbeing or "well being" or well-being or Anxiety or stress or depress* or lonely or loneliness or Self-harm or "self harm" or Self-esteem or "self esteem" or confidence or resilience or coping or flourishing or "quality of life" or "life satisfaction" or "positive affect" or "negative affect").ab. or (((mental or emotional or psychological or psychiatric or internalising or internalizing or anxiety or depressive) and (health or illness* or disorder* or symptom* or distress or difficult*)) or Wellbeing or "well being" or well-being or Anxiety or stress or depress* or lonely or loneliness or Self-harm or "self harm" or Self-esteem or "self esteem" or confidence or resilience or coping or flourishing or "quality of life" or "life satisfaction" or "positive affect" or "negative affect").ti. |
| Associations | 4 | (Associat* or relationship or related or effect or causal or link or influence or impact*).ab. or (Associat* or relationship or related or effect or causal or link or influence or impact*).ti. |
| Quantitative study design | 5 | (Quantitative or Empiric* or Observation* or epidemiolog* or "cross-sectional" or Longitudinal or "case-control" or Ecological or Multilevel or "multi level" or "multi-level").af. |
|  | 6 | 1 and 2 and 3 and 4 and 5 |
|  | 7 | 6 and 2014:2024.(sa_year) |

**Appendix 5. JBI Critical Appraisal and Bradford Hill Viewpoint scores**

Table A5. Summary of JBI critical appraisal scores and BH viewpoint ratings, by result

| **First author, year** | **Outcome category** | **Direction of result** | **Arts category** | **Sub category** | **JBI Critical Appraisal Score (%)** | **JBI Rating (Weak, Moderate, Strong)** | **BH Strength** | **BH Temporality** | **BH Dose-response** | **BH Experiment** |
| --- | --- | --- | --- | --- | --- | --- | --- | --- | --- | --- |
| Badura, 2015 | EXT | Null | Multiple / not separated |  | 3.5/7 (50%) | MODERATE | Weak | Weak | Weak | Weak |
| Badura, 2015 | INT | Null | Multiple / not separated |  | 3.5/7 (50%) | MODERATE | Weak | Weak | Weak | Weak |
| Badura, 2015 | WB | Pos | Multiple / not separated |  | 3.5/7 (50%) | MODERATE | Moderate | Weak | Weak | Weak |
| Bickham, 2015 | INT | Null | Music | Listening | 6/9 (67%) | MODERATE | Weak | Strong | Strong | Moderate |
| Bone, 2023 | INT | Null | Multiple / not separated |  | 7/9 (78%) | STRONG | Weak | Strong | Moderate | Moderate |
| Bone, 2023 | WB | Pos | Multiple / not separated |  | 7/9 (78%) | STRONG | Moderate | Strong | Moderate | Moderate |
| Bone, 2022 | EXT | Pos | Multiple / not separated |  | 6/9 (67%) | MODERATE | Moderate | Strong | Strong | Moderate |
| Clarke, 2018 | WB | Mixed | Other |  | 2.5/7 (36%) | WEAK | Moderate | Weak | Weak | Weak |
| da Silva, 2023 | EXT | Pos | Music | Listening | 3.5/7 (50%) | MODERATE | Moderate | Weak | Strong | Weak |
| Deer, 2023 | SP | Pos | Multiple / not separated |  | 4.5/7 (64%) | MODERATE | Moderate | Weak | Strong | Weak |
| Dorris, 2022 | SP | Null | Music | Both | 2.5/7 (36%) | WEAK | Weak | Weak | Weak | Weak |
| Felsman, 2019 (CH 2) | SP | Mixed | Drama |  | 6/9 (67%) | MODERATE | Strong | Strong | Weak | Weak |
| Felsman, 2019 (CH 4) | SP | Null | Drama |  | 7.5/9 (83%) | STRONG | Weak | Strong | Weak | Weak |
| Felsman, 2019 (CH 4) | INT | Pos | Drama |  | 7/9 (78%) | STRONG | Strong | Strong | Weak | Weak |
| Felsman, 2019 (CH 4) | INT | Pos | Drama |  | 7.5/9 (83%) | STRONG | Strong | Strong | Weak | Weak |
| Fluharty, 2023 | EXT | Pos | Multiple / not separated |  | 7.5/9 (83%) | STRONG | Moderate | Strong | Strong | Moderate |
| Foster, 2017 | EXT | Null | Music | Playing | 9/11 (82%) | STRONG | Weak | Strong | Weak | Strong |
| Foster, 2017 | SP | Null | Music | Playing | 9/11 (82%) | STRONG | Weak | Strong | Weak | Strong |
| Gottfried, 2021 | INT | Neg | Music | Playing | 6/7 (86%) | STRONG | Moderate | Weak | Weak | Weak |
| Gottfried, 2021 | INT | Pos | Other | Yearbook | 6/7 (86%) | STRONG | Moderate | Weak | Weak | Weak |
| Han, 2020 | INT | Pos | Music | Listening | 6/13 (46%) | WEAK | Strong | Strong | Weak | Strong |
| Huang, 2023 | INT | Pos | Music | Listening | 7/9 (78%) | STRONG | Moderate | Strong | Strong | Moderate |
| Huang, 2023 | INT | Pos | Music | Listening | 7/9 (78%) | STRONG | Moderate | Strong | Strong | Moderate |
| Huang, 2023 | INT | Pos | Music | Listening | 7/9 (78%) | STRONG | Moderate | Strong | Strong | Moderate |
| Kennewell, 2022 | WB | Mixed | Music | Playing | 5/7 (71%) | MODERATE | Moderate | Weak | Strong | Weak |
| Kennewell, 2022 | WB | Pos | Arts and Crafts |  | 5/7 (71%) | MODERATE | Moderate | Weak | Strong | Weak |
| Kwak, 2018 | INT | Neg | Multiple / not separated |  | 8/9 (89%) | STRONG | Weak | Weak | Moderate | Moderate |
| Kwak, 2018 | EXT | Null | Multiple / not separated |  | 8/9 (89%) | STRONG | Moderate | Weak | Moderate | Moderate |
| Kwak, 2018 | INT | Null | Multiple / not separated |  | 8/9 (89%) | STRONG | Weak | Weak | Moderate | Moderate |
| Kwon, 2020 | WB | Pos | Music | Listening | 6/9 (67%) | MODERATE | Moderate | Strong | Strong | Moderate |
| Mak, 2019 | SP | Pos | Music | Both | 6.5/7 (93%) | STRONG | Moderate | Weak | Moderate | Moderate |
| Mak, 2019 | SP | Pos | Other | Reading | 6.5/7 (93%) | STRONG | Moderate | Weak | Moderate | Moderate |
| Mak, 2019 | SP | Pos | Arts and Crafts |  | 6.5/7 (93%) | STRONG | Strong | Weak | Moderate | Moderate |
| McGrath, 2019 | WB | Null | Other | Circus Arts | 7/8 (88%) | STRONG | Weak | Strong | Weak | Weak |
| McGrath, 2019 | WB | Null | Other | Circus Arts | 7/8 (88%) | STRONG | Weak | Strong | Weak | Weak |
| Miranda, 2022 | INT | Neg | Music | Playing | 3/7 (43%) | WEAK | Weak | Weak | Moderate | Weak |
| O'Flaherty, 2022 | WB | Pos | Multiple / not separated |  | 8.5/11 (77%) | STRONG | Weak | Weak | Weak | Strong |
| O'Flaherty, 2022 | EXT | Pos | Multiple / not separated |  | 8.5/11 (77%) | STRONG | Weak | Weak | Weak | Strong |
| O'Flaherty, 2022 | INT | Pos | Multiple / not separated |  | 8.5/11 (77%) | STRONG | Weak | Weak | Weak | Strong |
| O'Flaherty, 2022 | INT | Pos | Multiple / not separated |  | 8.5/11 (77%) | STRONG | Moderate | Weak | Weak | Strong |
| O'Flaherty, 2022 | WB | Pos | Multiple / not separated |  | 8.5/11 (77%) | STRONG | Weak | Weak | Weak | Strong |
| Oosterhof, 2017 | ADD | Pos | Music | Playing | 6.5/7 (93%) | STRONG | Moderate | Weak | Strong | Weak |
| Oosterhof, 2017 | SP | Pos | Music | Playing | 6.5/7 (93%) | STRONG | Moderate | Weak | Strong | Weak |
| Oosterhof, 2017 | ADD | Pos | Other | Yearbook | 6.5/7 (93%) | STRONG | Weak | Weak | Strong | Weak |
| Oosterhof, 2017 | SP | Pos | Other | Yearbook | 6.5/7 (93%) | STRONG | Weak | Weak | Strong | Weak |
| Pongutta, 2023 | WB | Neg | Music | Both | 5/7 (71%) | MODERATE | Moderate | Weak | Moderate | Weak |
| Pongutta, 2023 | WB | Null | Arts and Crafts |  | 5/7 (71%) | MODERATE | Weak | Weak | Moderate | Weak |
| Ros-Morente, 2019 | WB | Pos | Music | Playing | 3.5/7 (50%) | MODERATE | Strong | Weak | Weak | Weak |
| Ros-Morente, 2019 | WB | Pos | Music | Playing | 3.5/7 (50%) | MODERATE | Strong | Weak | Weak | Weak |
| Saramento, 2024 | WB | Pos | Other | Online Radio | 7/9 (78%) | STRONG | Moderate | Strong | Moderate | Strong (to moderate) |
| Swarmi, 2018 | SP | Null | Arts and Crafts |  | 6/8 (75%) | STRONG | Strong | Strong | Weak | Weak |
| Yeo, 2023 | SP | Null | Multiple / not separated |  | 8/22 (73%) | MODERATE | Weak | Strong | Weak | Moderate |
| Yıldırım, 2024 | ADD | Pos | Multiple / not separated |  | 5/7 (71%) | MODERATE | Moderate | Weak | Weak | Weak |
| Yıldırım, 2024 | EXT | Pos | Multiple / not separated |  | 5/7 (71%) | MODERATE | Moderate | Weak | Weak | Weak |
| Yıldırım, 2024 | WB | Pos | Multiple / not separated |  | 5/7 (71%) | MODERATE | Moderate | Weak | Weak | Weak |

**Note:** WB = Positive wellbeing indicators, INT = Internalising mental health symptoms, EXT = Externalising mental health symptoms, SP = Self-perception, ADD = Addictive behaviours. Pos = Positive, Neg = Negative

**Appendix 6. GRADE Summary of Findings**

Table A6

| **GRADE Domain** | **Judgement** | **Concerns about certainty domains** |
| --- | --- | --- |
| Methodological limitations | The vast majority of included studies (24/28) were rated ‘Strong’ or ‘Moderate’ according to JBI critical appraisal tools. ‘Weak’ studies were generally spread across all directions of results. | Not serious |
| Indirectness | The populations of interest, exposures, and outcomes in included studies all provide direct evidence for the question at hand. | Not serious |
| Imprecision | Sample sizes varied dramatically, but some positive results came from studies with very large sample sizes. However, there were a number of studies with null, negative, and mixed results. | Not serious, borderline |
| Inconsistency | The direction and significance of results varied across studies. However, more than half of significant results suggested positive associations, and these were found in a variety of settings. | Not serious, borderline |
| Publication bias | Although formal analysis of publication bias was not undertaken, included studies found positive, negative, and null results. The search for evidence was comprehensive, and included unpublished research such as pre-prints, working papers, doctoral dissertations, and grey literature. | Not suspected |

**Appendix 7. Additional plots by arts activity and outcome category**

Figure A1. Plots of support for Bradford Hill viewpoints, by activity


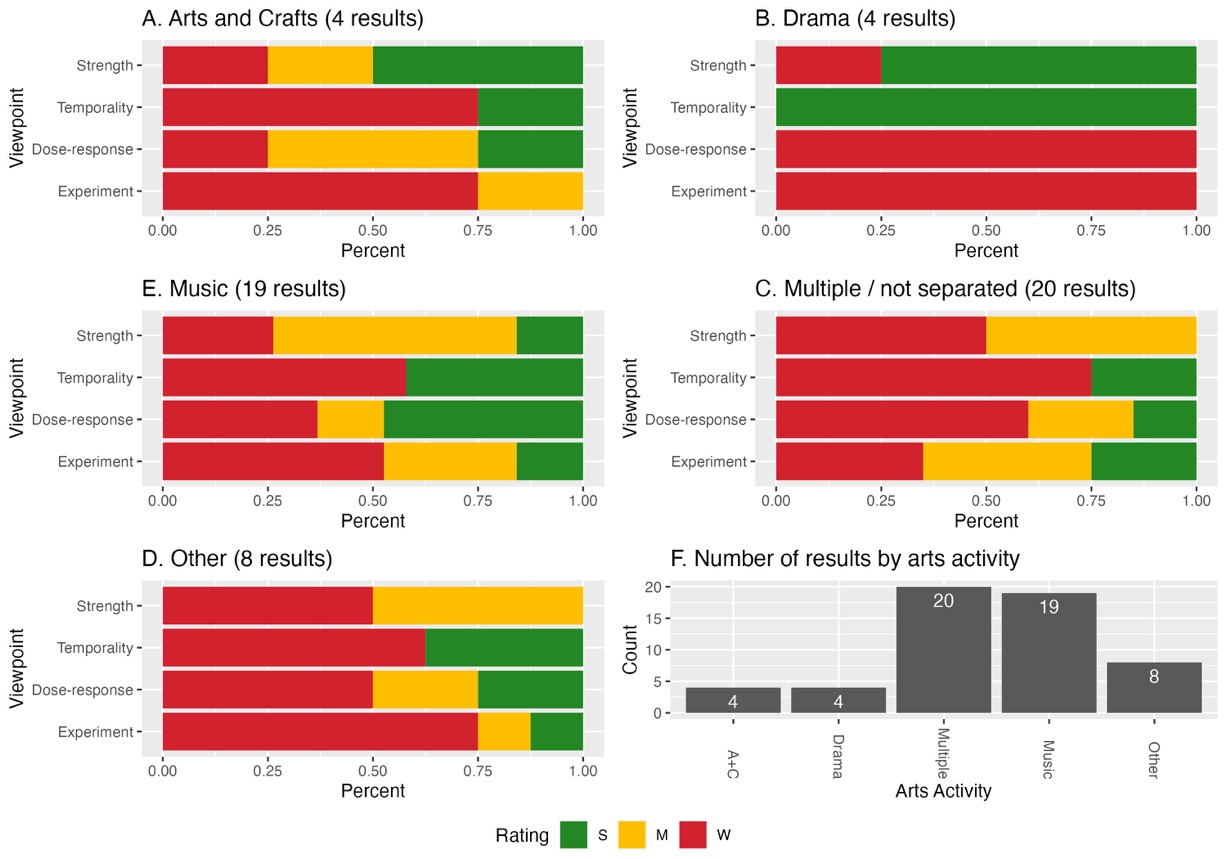


Figure A2. Plots of support for Bradford Hill viewpoints, by activity (music sub-categories)


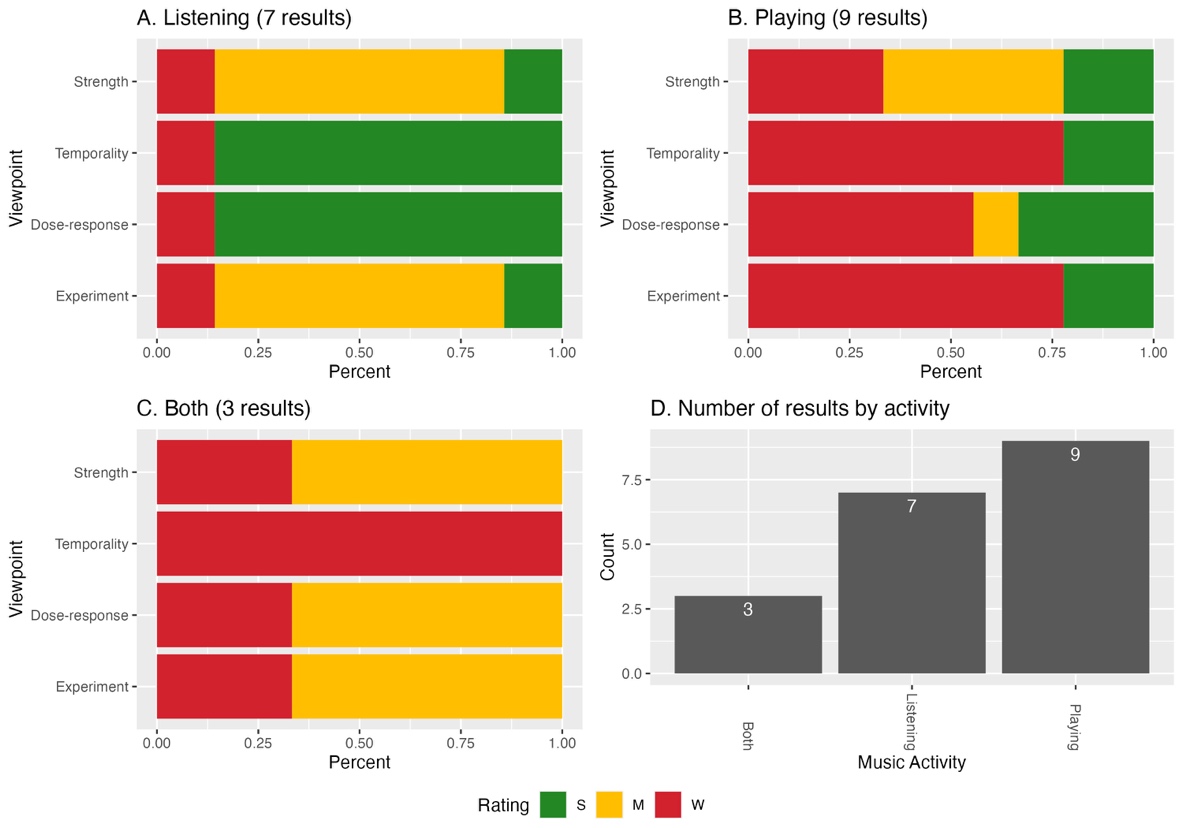


Figure A3. Plots of support for Bradford Hill viewpoints, by outcome


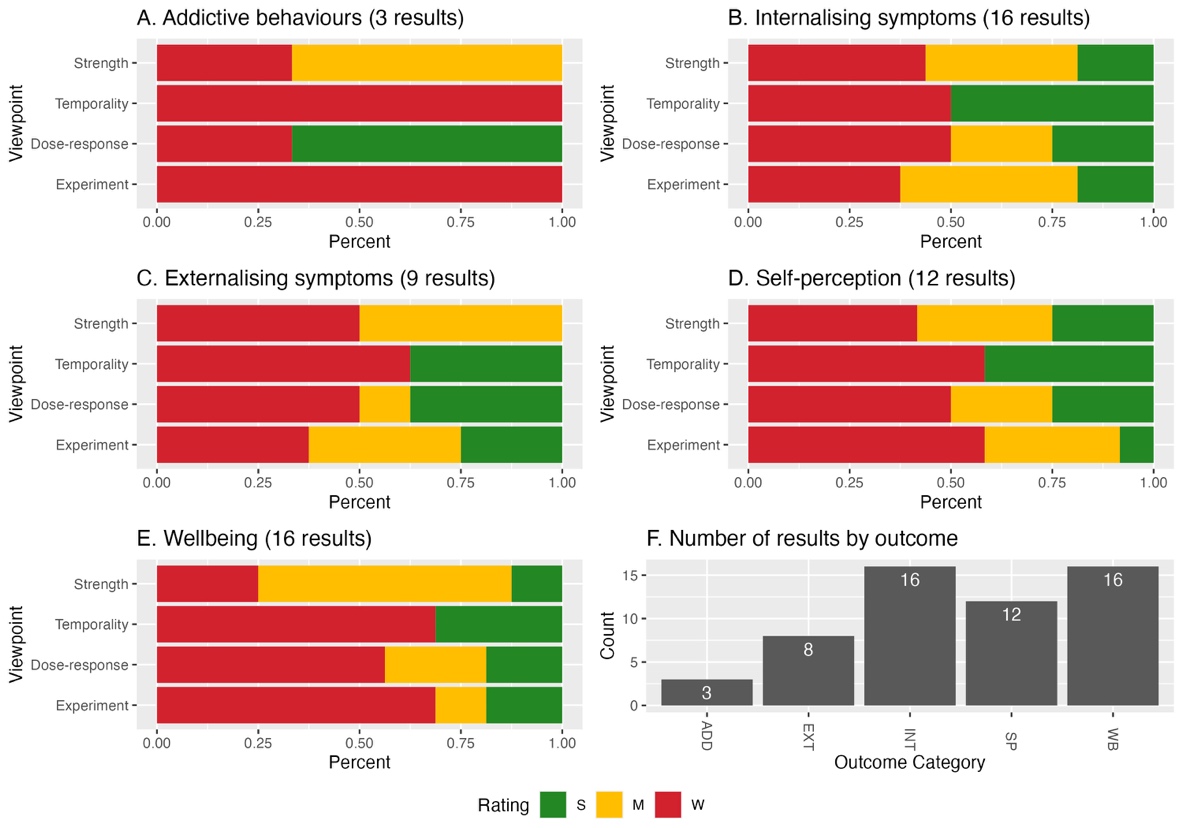


**Note:** WB = Positive wellbeing indicators, INT = Internalising mental health symptoms, EXT = Externalising mental health symptoms, SP = Self-perception, ADD = Addictive behaviours

Harvest plots by activity

Figure A4. Arts and crafts


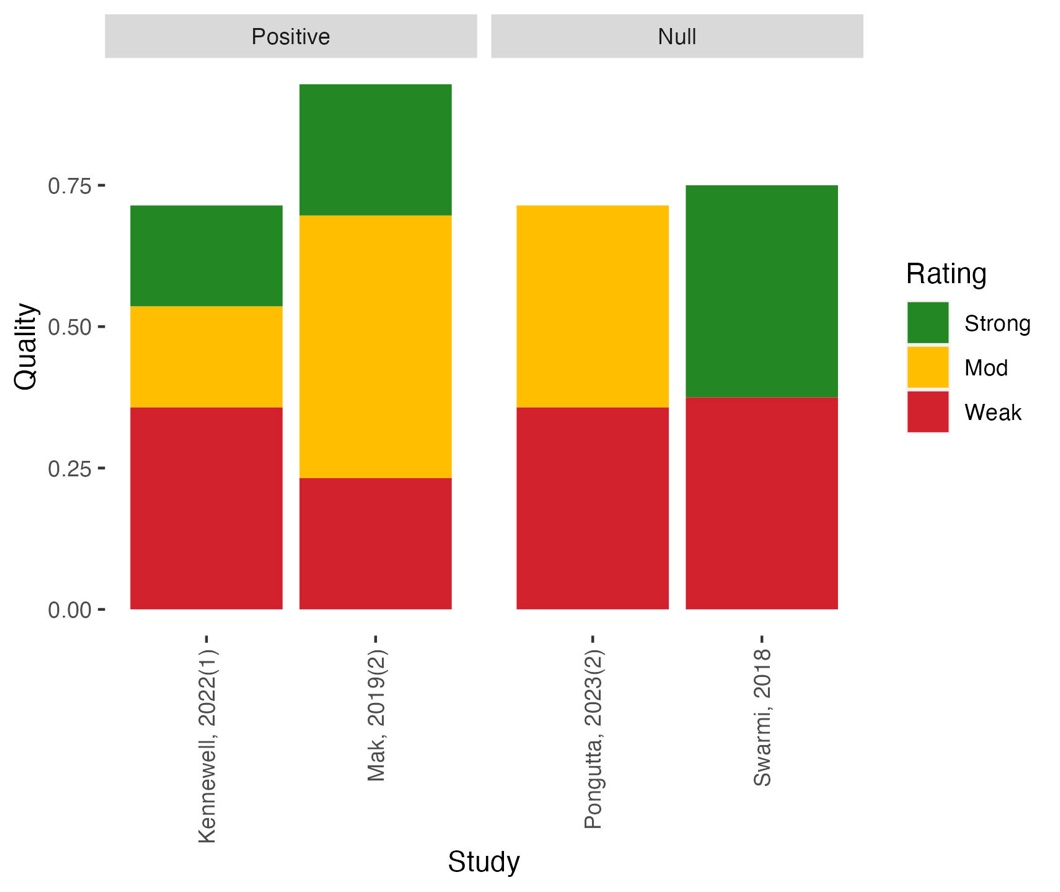


Figure A5. Drama


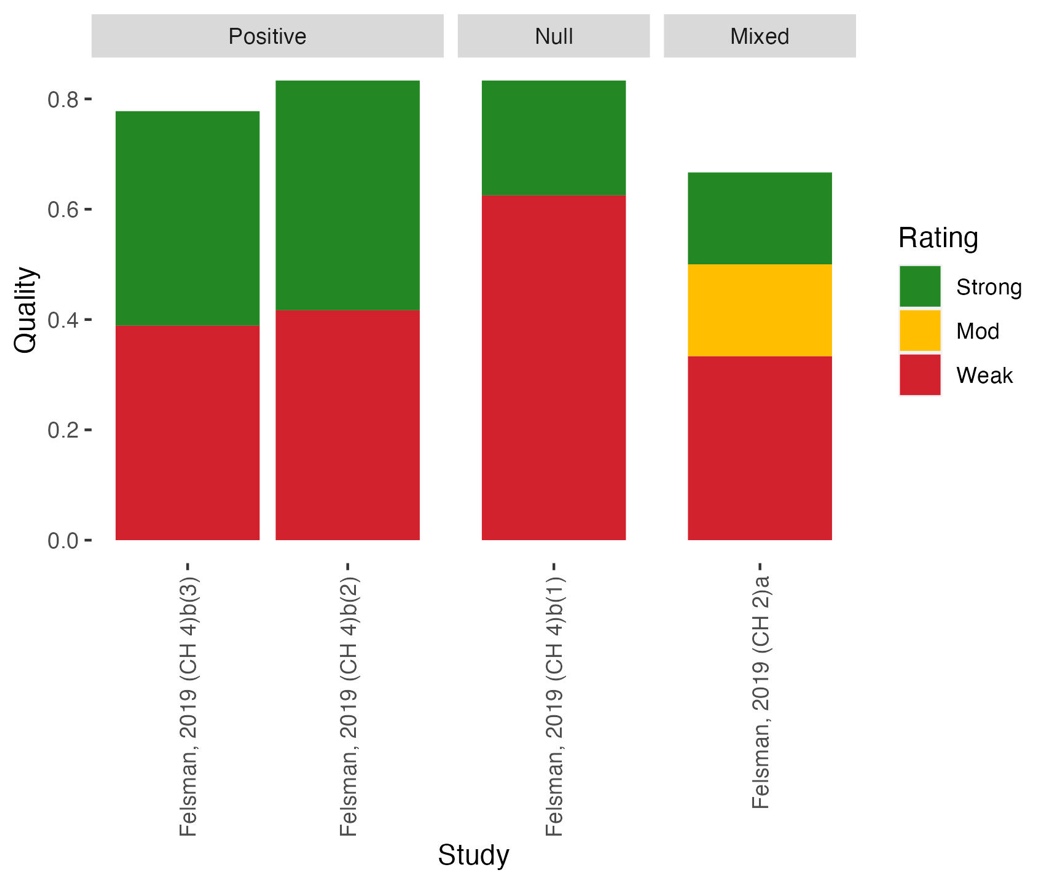


Figure A6. Music


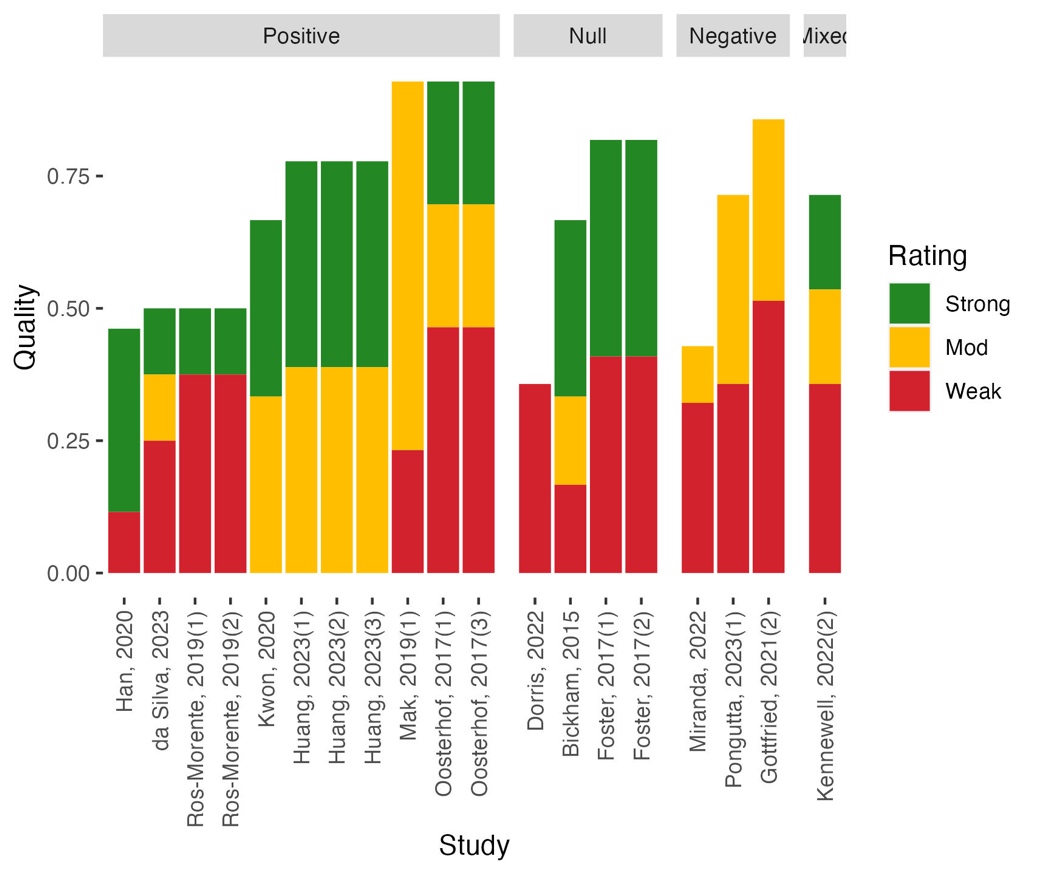


Figure A7. Other


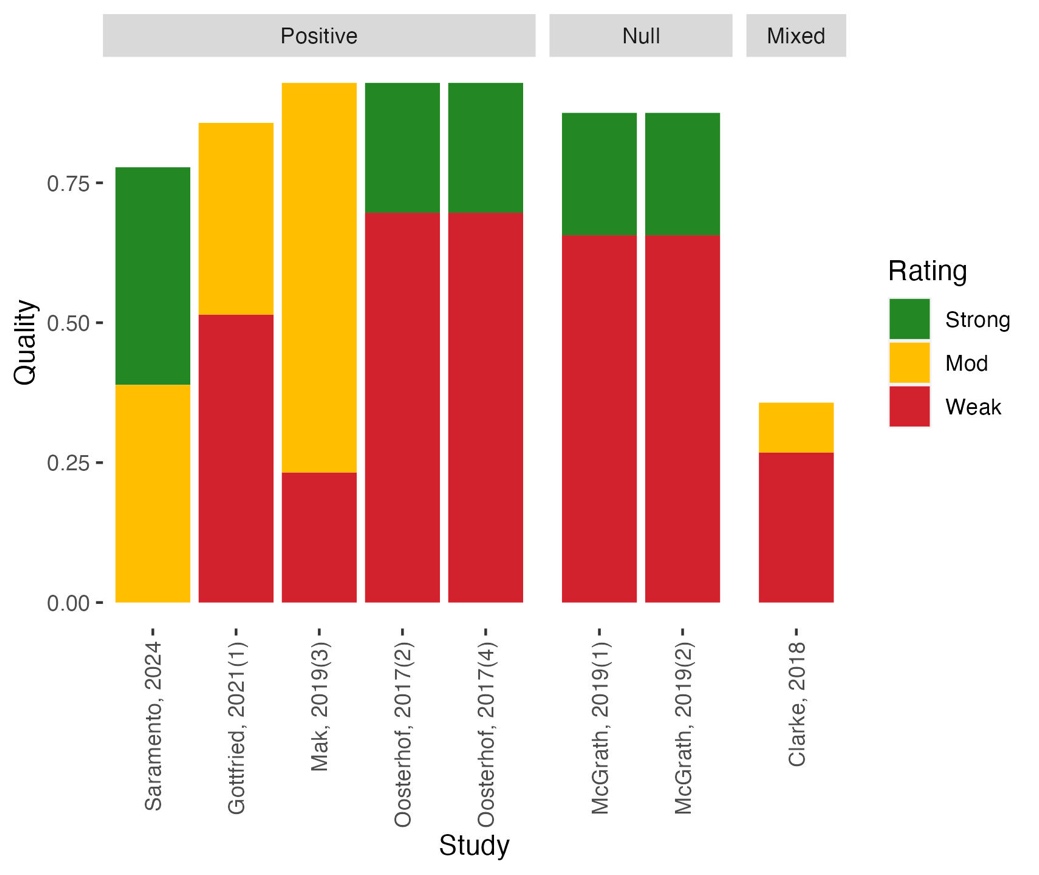


Figure A8. Multiple


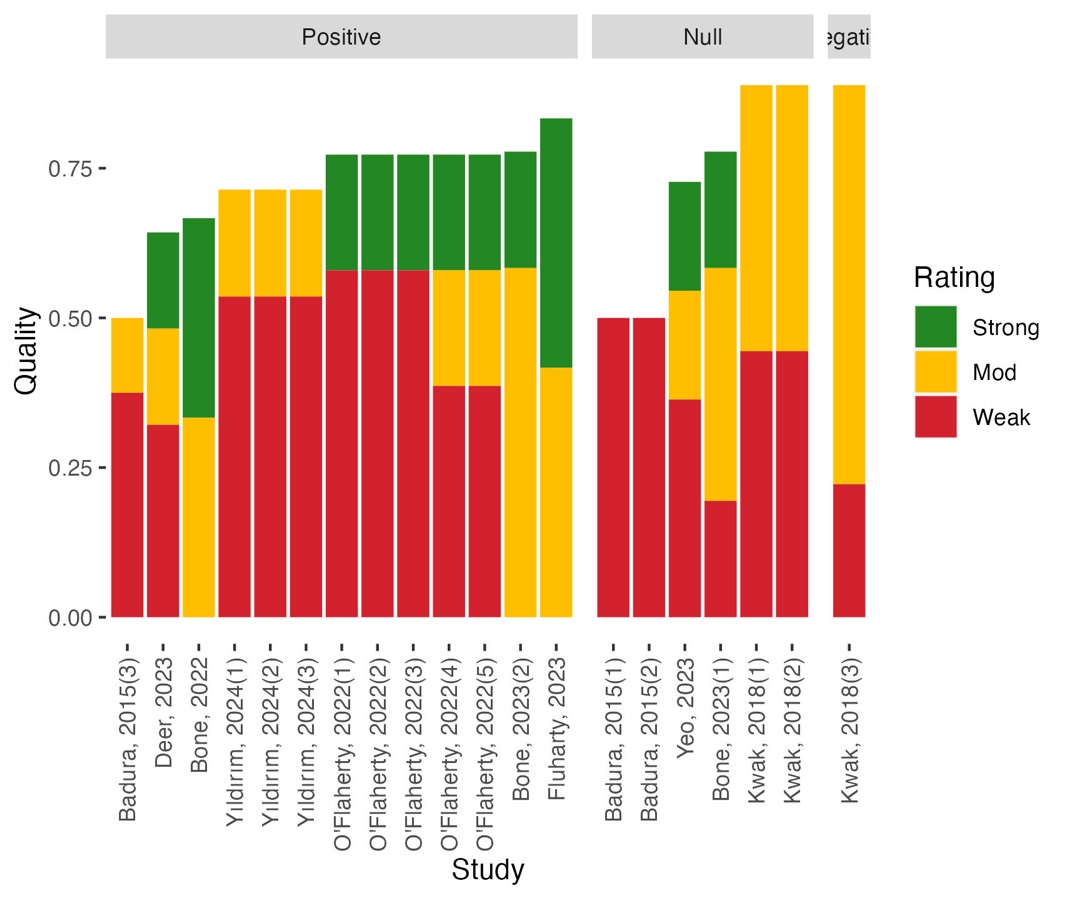


Figure A9. Music (listening)


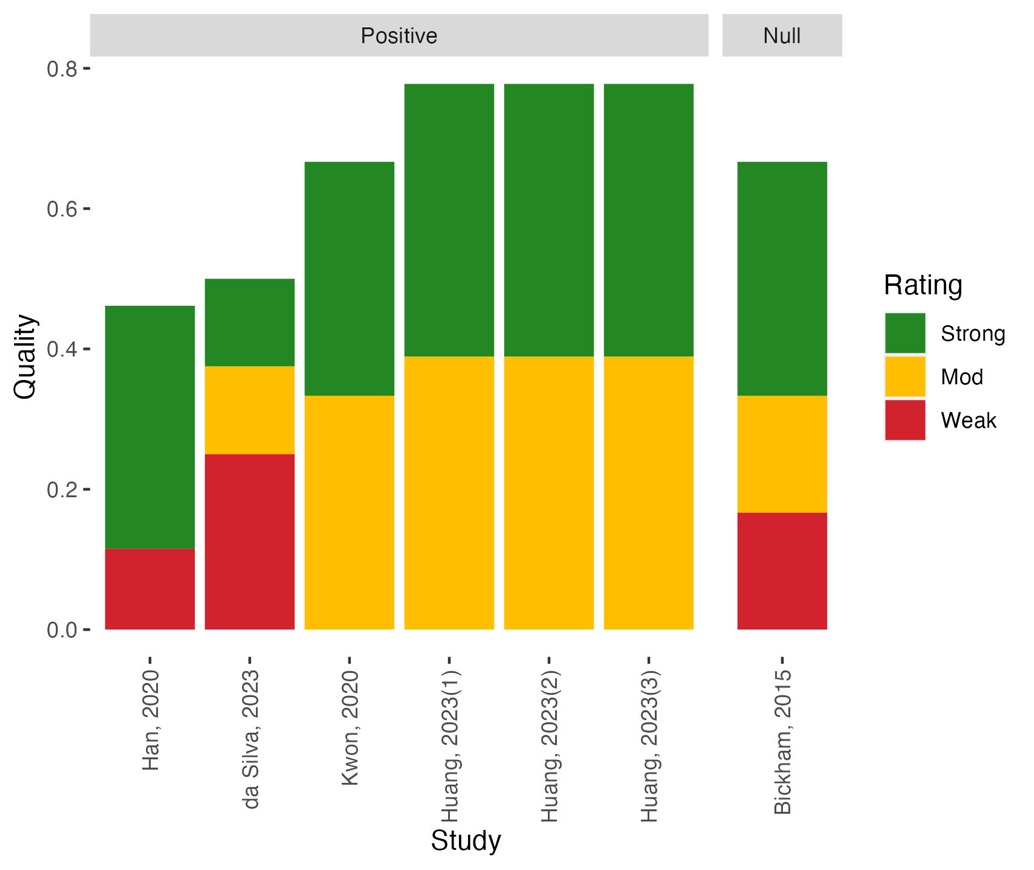


Figure A10. Music (playing)


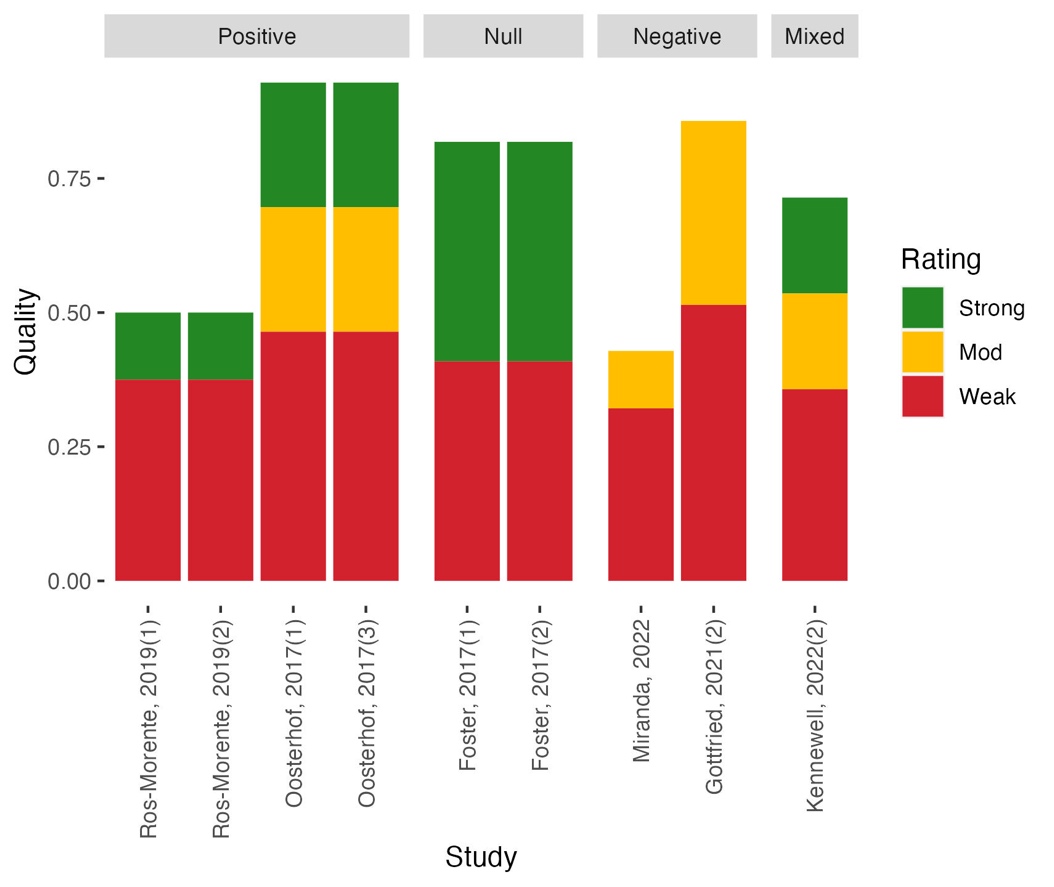


Figure A11. Music (both)


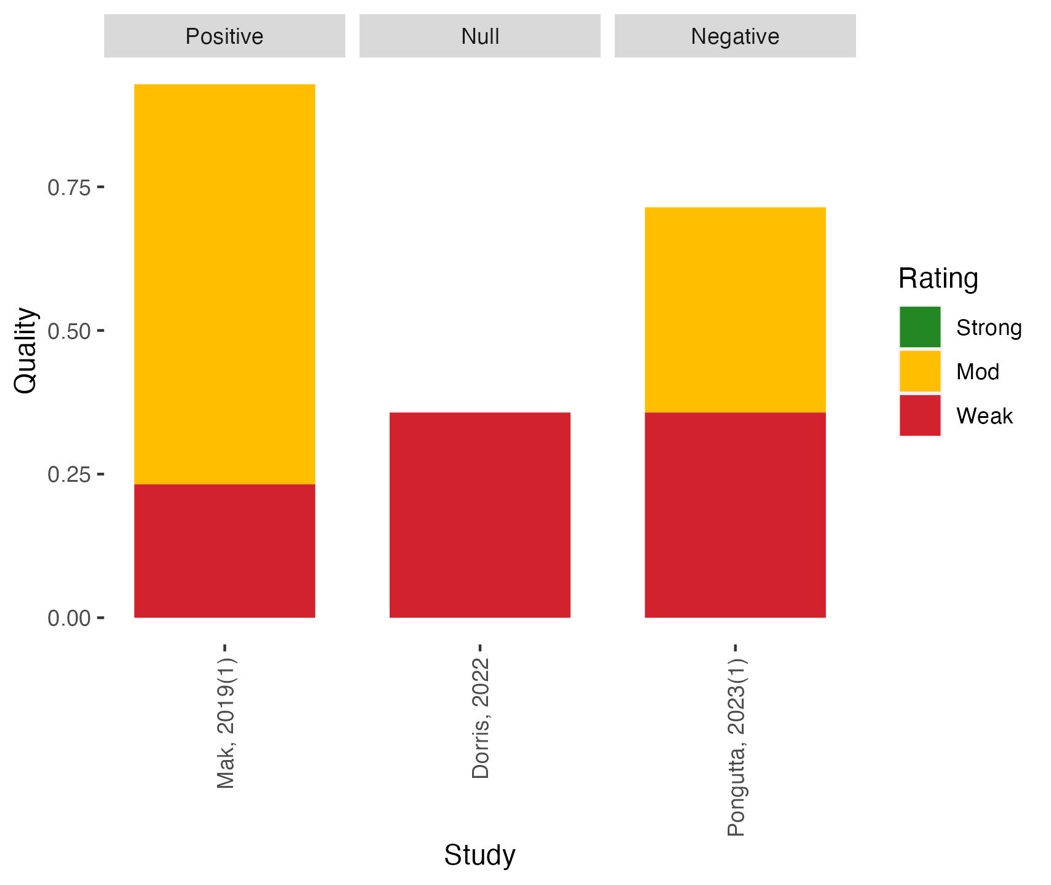


Harvest plots by outcome

Figure A12. Addictive behaviours


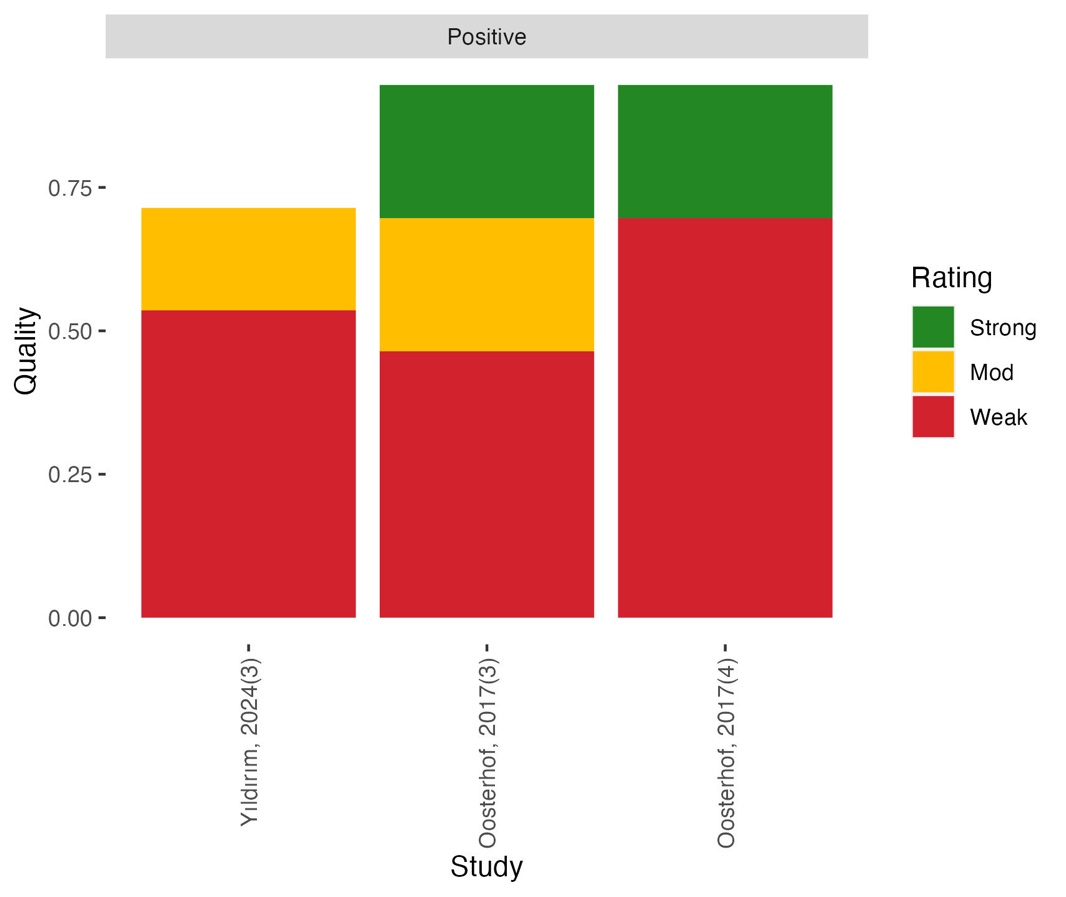


Figure A13. Externalising symptoms


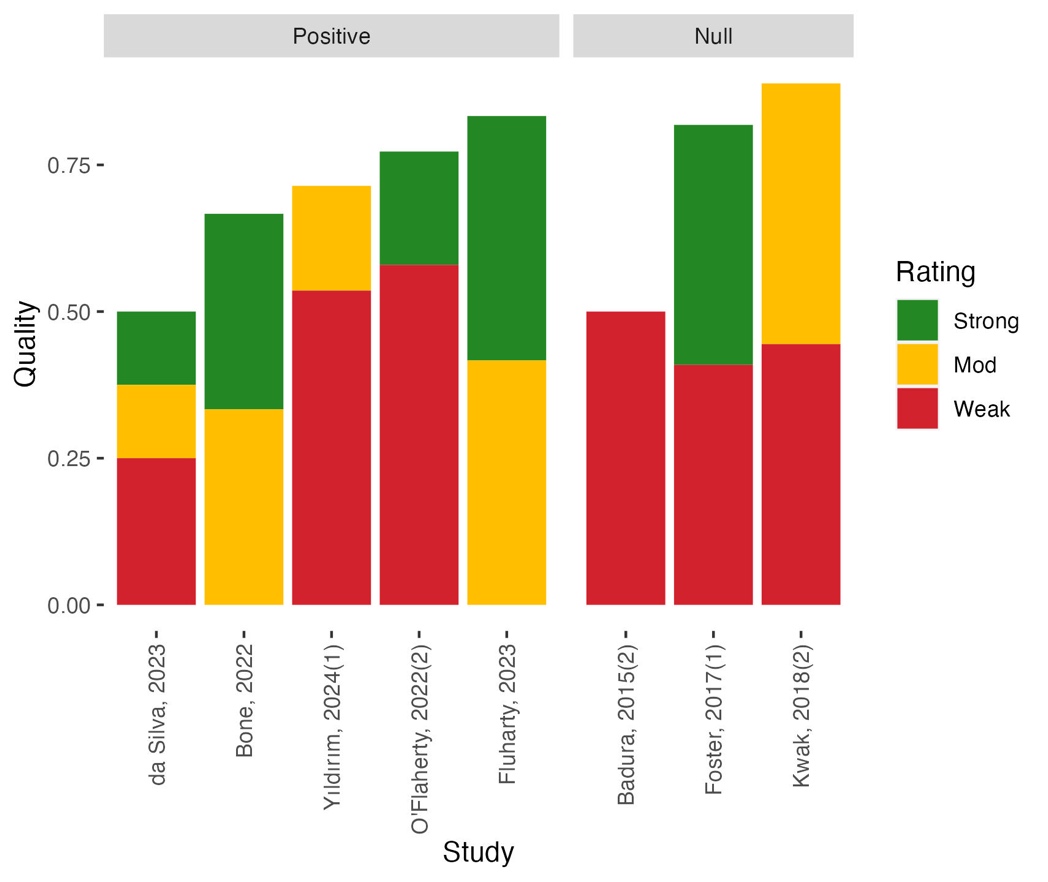


Figure A14. Internalising Symptoms


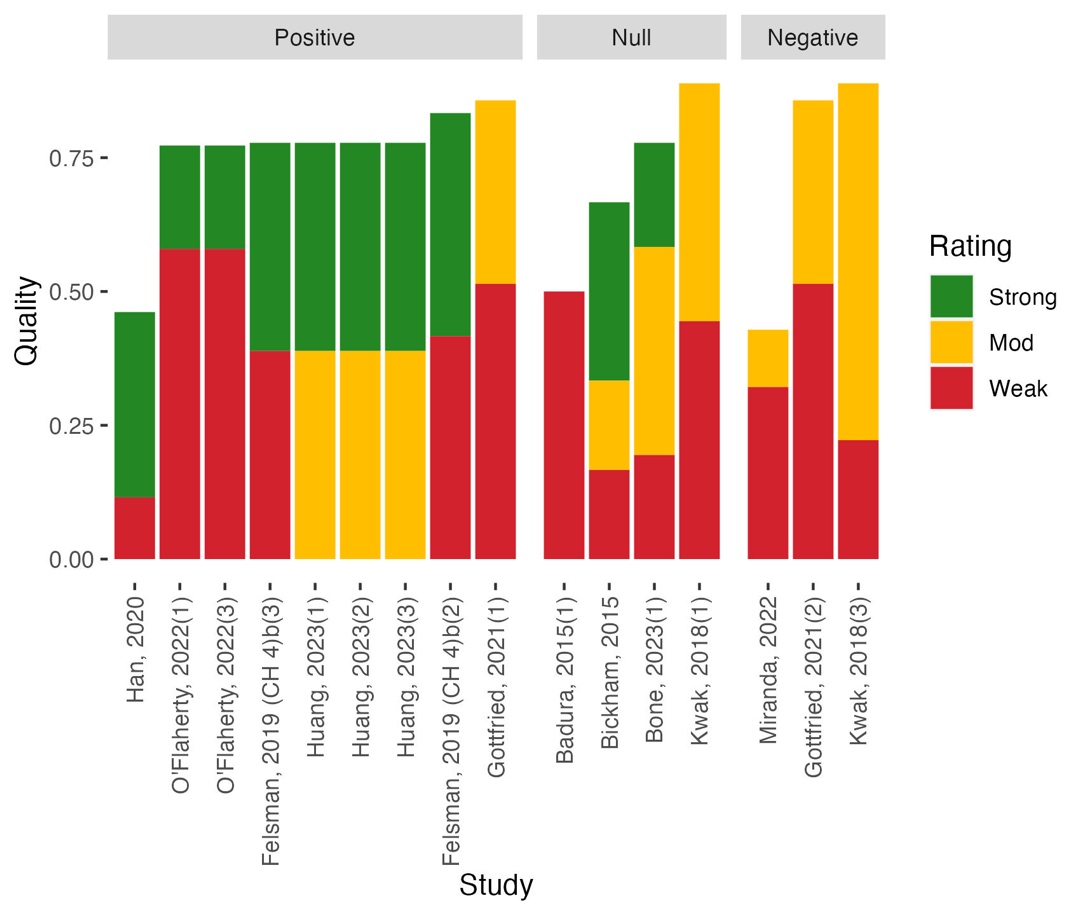


Figure A15. Self-perception


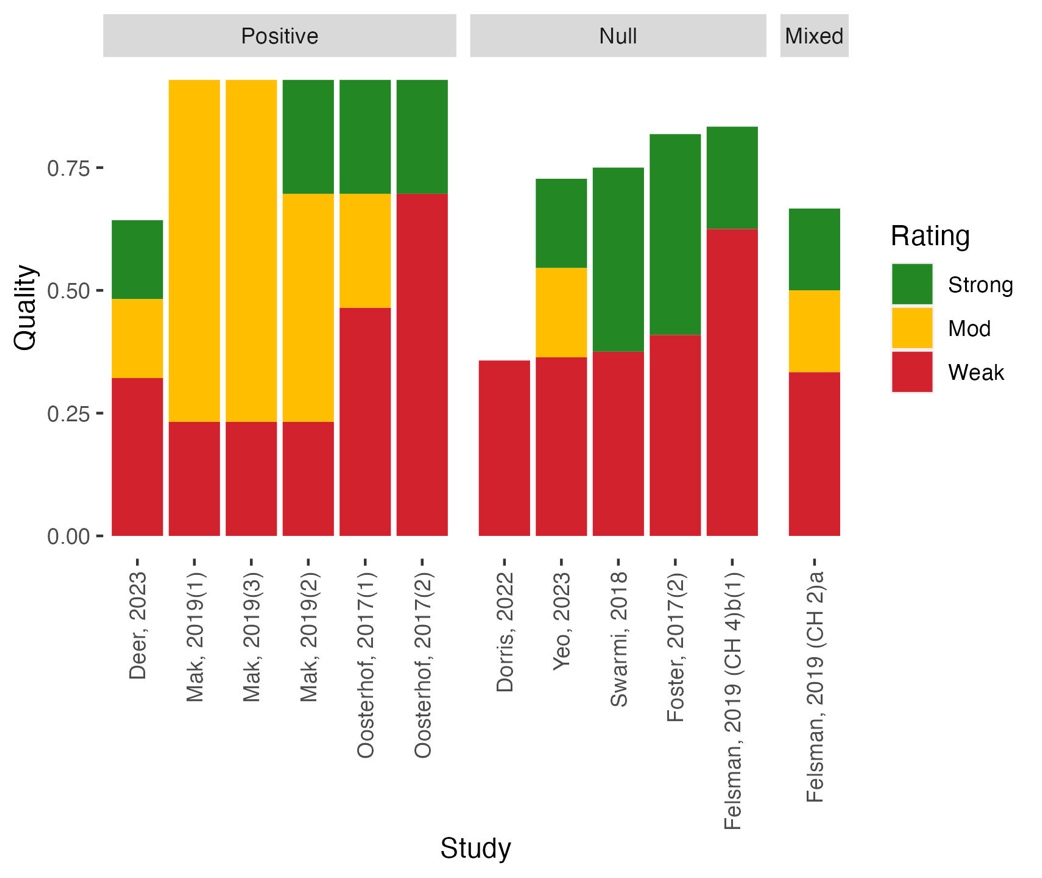


Figure A16. Wellbeing


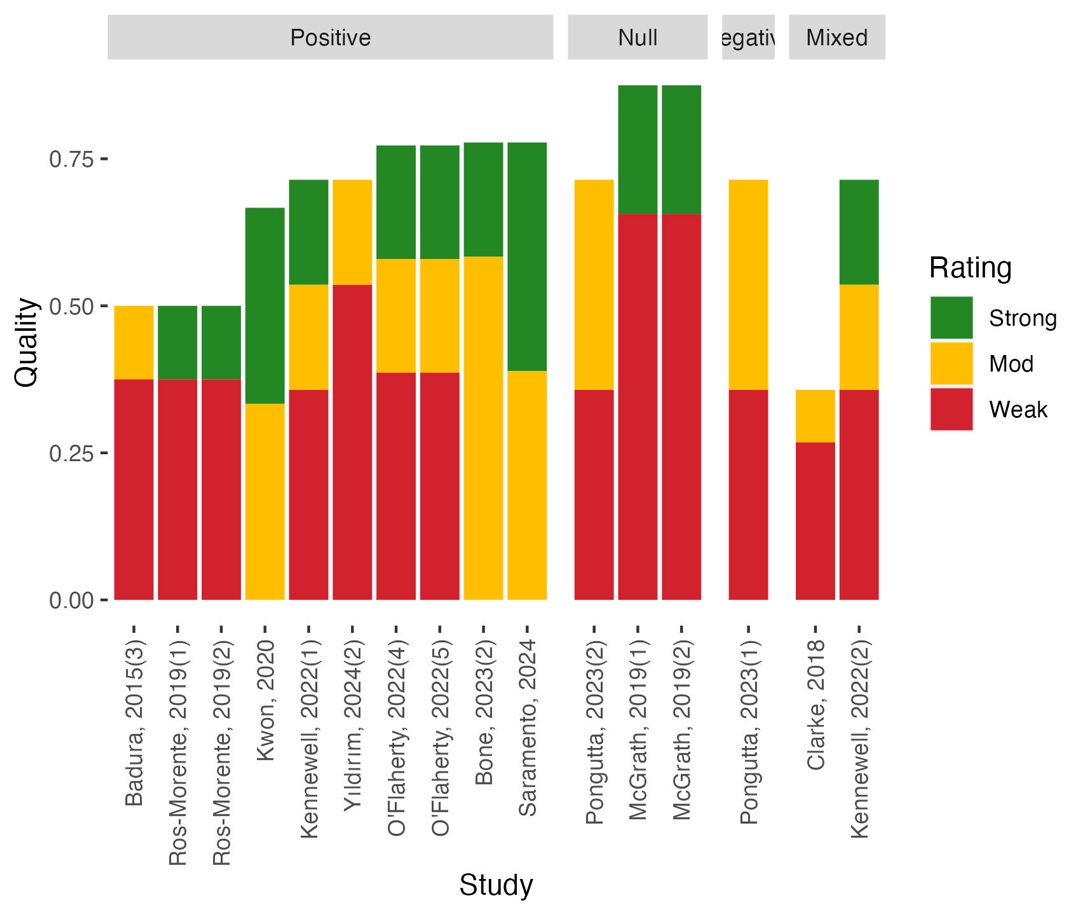


**Additional Appendix References**

Akoglu, H. (2018). User’s guide to correlation coefficients. *Turkish Journal of Emergency Medicine*, *18*(3), 91–93. <https://doi.org/10.1016/j.tjem.2018.08.001>

Cohen, J. (2013). *Statistical Power Analysis for the Behavioral Sciences* (2nd ed.). Routledge. <https://doi.org/10.4324/9780203771587>

de Vocht, F., Katikireddi, S. V., McQuire, C., Tilling, K., Hickman, M., & Craig, P. (2021). Conceptualising natural and quasi experiments in public health. *BMC Medical Research Methodology*, *21*(1), 32. <https://doi.org/10.1186/s12874-021-01224-x>

Livesey, G., Taylor, R., Livesey, H. F., Buyken, A. E., Jenkins, D. J. A., Augustin, L. S. A., Sievenpiper, J. L., Barclay, A. W., Liu, S., Wolever, T. M. S., Willett, W. C., Brighenti, F., Salas-Salvadó, J., Björck, I., Rizkalla, S. W., Riccardi, G., Vecchia, C. la, Ceriello, A., Trichopoulou, A., … Brand-Miller, J. C. (2019). Dietary Glycemic Index and Load and the Risk of Type 2 Diabetes: Assessment of Causal Relations. *Nutrients*, *11*(6), 1436. <https://doi.org/10.3390/nu11061436>

Munn, Z., Porritt, K., Aromataris, E., Lockwood, C., & Peters, M. (2014). Supporting Document for the Joanna Briggs Institute Levels of Evidence and Grades of Recommendation. *Joanna Briggs Institute.* <https://jbi.global/sites/default/files/2019-05/JBI%20Levels%20of%20Evidence%20Supporting%20Documents-v2.pdf>

Pickett, K. E., & Wilkinson, R. G. (2015). Income inequality and health: A causal review. *Social Science & Medicine*, *128*, 316–326. <https://doi.org/10.1016/j.socscimed.2014.12.031>

Roffey, D. M., Wai, E. K., Bishop, P., Kwon, B. K., & Dagenais, S. (2010). Causal assessment of workplace manual handling or assisting patients and low back pain: results of a systematic review. *The Spine Journal*, *10*(7), 639–651. <https://doi.org/10.1016/j.spinee.2010.04.028>

Sullivan, G. M., & Feinn, R. (2012). Using Effect Size—or Why the *P* Value Is Not Enough. *Journal of Graduate Medical Education*, *4*(3), 279–282. <https://doi.org/10.4300/JGME-D-12-00156.1>
